# Estimated impact of maternal vaccination on global paediatric influenza-related in-hospital mortality: A retrospective case series

**DOI:** 10.1016/j.eclinm.2021.100945

**Published:** 2021-06-10

**Authors:** Yvette N Löwensteyn, Harish Nair, Marta C Nunes, Ichelle van Roessel, Femke S Vernooij, Joukje Willemsen, Louis J Bont, Natalie I Mazur, FLU GOLD study group, Kentigern Thorburn, Marta Nunes, Richard Chawana, Shabir A. Madhi, Anna C. Vittuci, Quique Bassat, Azucena Bardají, Edward Goka, Srđan Roglić, Bosco Paes, LouAnn Elliott, Hitoshi Oshitani, Socorro Lupisan, Angela Gentile, María Florencia Lucion, Michael C. Spaeder, Warwick Butt, Jenny Thompson, Asuncion Mejias, Octavio Ramilo, Rodrigo A. Fasce, Marta Werner, Diego R. Hijano, Kim J. Allison, Márcia Rosane Pires, Fernanda de-Paris, Giorgi Chakhunashvili, Irakli Karseladze, Grieven P. Otieno, D. James Nokes, Oded Scheuerman, Dario Prais, Mohammed Al Amad, Abdul Wahed Al Serouri, Asad Ali, Mohammad Tahir Yousafzai, Alfredo Bruno, Domenica de Mora, Jenny Ojeda, Ghassan Dbaibo, Rima Hanna-Wakim, Vassiliki Papaevangelou, Elpiniki Kartisouni, Cheryl Cohen, Sibongile Walaza, Rosalie S. Linssen, Hsin Chi, Aykut Eşki, Esen Demir, Senjuti Saha, Samir K Saha, Anthony A. Sochet, Beatriz E. Teppa-Sanchez, Thyyar M. Ravindranath, J. Scott Baird, Shaun K. Morris, Waison Wong, Robert F. Breiman, Emily S. Gurley, Shams El Arifeen, Nega Assefa, J. Anthony G. Scott, Dickens Onyango, Karen L. Kotloff, Samba O. Sow, Inacio Mandomando, Ikechukwu U. Ogbuanu, Amara Jambai, Tanil Kendirli, Edin Botan, Franco Díaz Rubio, Alberto Serra, Luis Martinez, Luis Pedroso, Soledad Menta, Rosalba Pardo, Alejandro Donoso, Syed Faisal Mahmood, Naveera Khan

**Affiliations:** a Department of Paediatric Infectious Diseases and Immunology, Wilhelmina Children's Hospital, University Medical Centre Utrecht, Utrecht, the Netherlands; b Respiratory Syncytial Virus Network (ReSViNET) Foundation, Zeist, the Netherlands; c Centre for Global Health, Usher Institute, Edinburgh Medical School, University of Edinburgh, Edinburgh, UK; d South African Medical Research Council: Vaccines and Infectious Diseases Analytics Research Unit, School of Pathology, Faculty of Health Sciences, University of the Witwatersrand; and Department of Science and Technology/National Research Foundation: Vaccine Preventable Diseases Unit, University of the Witwatersrand, Johannesburg, South Africa

## Abstract

**Background:**

Influenza virus infection is an important cause of under-five mortality. Maternal vaccination protects children younger than 3 months of age from influenza infection. However, it is unknown to what extent paediatric influenza-related mortality may be prevented by a maternal vaccine since global age-stratified mortality data are lacking.

**Methods:**

We invited clinicians and researchers to share clinical and demographic characteristics from children younger than 5 years who died with laboratory-confirmed influenza infection between January 1, 1995 and March 31, 2020. We evaluated the potential impact of maternal vaccination by estimating the number of children younger than 3 months with in-hospital influenza-related death using published global mortality estimates.

**Findings:**

We included 314 children from 31 countries. Comorbidities were present in 166 (53%) children and 41 (13%) children were born prematurely. Median age at death was 8·6 (IQR 4·5–16·6), 11·5 (IQR 4·3–24·0), and 15·5 (IQR 7·4–27·0) months for children from low- and lower-middle-income countries (LMICs), upper-middle-income countries (UMICs), and high-income countries (HICs), respectively. The proportion of children younger than 3 months at time of death was 17% in LMICs, 12% in UMICs, and 7% in HICs. We estimated that 3339 annual influenza-related in-hospital deaths occur in the first 3 months of life globally.

**Interpretation:**

In our study, less than 20% of children is younger than 3 months at time of influenza-related death. Although maternal influenza vaccination may impact maternal and infant influenza disease burden, additional immunisation strategies are needed to prevent global influenza-related childhood mortality. The missing data, global coverage, and data quality in this study should be taken into consideration for further interpretation of the results.

**Funding:**

Bill & Melinda Gates Foundation.

**Research in Context**

**Evidence before this study**

Influenza infection was associated with an estimated 15,300 (uncertainty range [UR] 5800–43,800) in-hospital acute lower respiratory infection (ALRI) deaths in children younger than 5 years in 2018, of which 36% occurred in infants under 6 months. The potential impact of maternal vaccination on paediatric mortality is unclear as age-stratified data under 6 months at time of death are lacking and maternally-derived influenza antibodies provide protection up to the first 3 months of life. We searched for articles published in English from January 1, 2014 to February 2, 2020 using PubMed and search terms concerning influenza infection and childhood mortality. Publications originated from high-income countries (HICs) only and there were no multi-country case series.

**Added value of this study**

To our knowledge, this is the first global case series of children who died with laboratory-confirmed influenza infection reporting age-stratified data in young children. We provide clinical and demographic characteristics of 314 paediatric in-hospital deaths with laboratory-confirmed influenza infection from 31 countries. In our study, 91 children (29%) were from LMICs. Less than 20% of children dying in-hospital with influenza were younger than 3 months at time of death and children from LMICs were younger (8·6 months) than children from HICs (15·5 months) at time of death.

**Implications of all the available evidence**

Our data show that maternal vaccination may prevent a higher proportion of influenza-related in-hospital deaths in children in LMICs than in other World Bank income regions. However, even in LMICs, more than 80% of children are 3 months or older at time of influenza-related in-hospital death. This implies that a maternal vaccine could maximally prevent a small proportion of global paediatric influenza-related in-hospital mortality and other strategies are expected to have greater impact. By applying the proportions of children younger than 3 months at time of death to global mortality estimates, we calculated that maternal vaccination can potentially prevent up to 3339 (UR 1287–9886) influenza-related in-hospital deaths in children younger than 5 years annually. The missing data, global coverage, and data quality in this study should be taken into consideration for further interpretation of the results.

Alt-text: Unlabelled box

## 1 Introduction

Influenza virus is an important cause of mortality due to acute lower respiratory infection (ALRI) in young children. In 2018, influenza was associated with an estimated 15,300 (uncertainty range [UR] 5800–43,800) in-hospital deaths in children younger than 5 years of age globally [1]. More than one third of in-hospital deaths occurred in children younger than 6 months and the majority (82%) occurred in low-income (LICs) and lower-middle-income countries (LMICs) [1]. Children younger than 6 months of age have higher rates of influenza-associated hospitalisation and influenza-related mortality when compared to older children in high-income countries (HICs) [2,3].

Paediatric influenza vaccines are licensed for children aged 6 months and older [4]. This leaves a gap in protection during the first 6 months of life. Maternal vaccination has the potential to protect infants early in life through transplacental transfer of maternal antibodies against influenza virus. The World Health Organization (WHO) announced in 2012 that pregnant women should have the highest priority for seasonal influenza vaccination [5]. Since then, several countries have incorporated maternal influenza vaccination into routine immunisation programs, although implementation has been negligible in LMICs due to logistical challenges, vaccine acceptance, and costs [6]. In 2014, the Board of Gavi, the Vaccine Alliance, advocated for more evidence of vaccine impact on infants’ health outcome to support maternal influenza vaccination programmes in countries eligible for their support [7]. The efficacy of maternal influenza vaccination has been demonstrated in 4 randomised controlled trials, reducing laboratory-confirmed influenza in infants by 30–63% in the first 6 months of life [8], [9], [10], [11].

In the secondary analysis of a maternal influenza vaccine trial duration of protection by maternal vaccination was shown to be limited to the first 3 months of life due to the decline of maternally-derived antibodies [12]. However, it remains unknown to what extent maternal influenza vaccination could prevent influenza-related mortality in infants, as global granular data on age distribution under 6 months at time of death are lacking [13]. Studies examining vaccine efficacy against influenza-related mortality from countries where maternal vaccination has been implemented exclude children younger than 6 months of age or do not collect maternal vaccination data [2,13,14]. Thus, the potential impact of maternal influenza vaccination on paediatric influenza-related mortality remains unknown.

To gain insight into the age distribution and clinical characteristics of influenza-related mortality in young children worldwide, we initiated the FLU Global Online Mortality Database (FLU GOLD) study. Using retrospective data, we determined the characteristics and age distribution of children younger than 5 years who died in-hospital with laboratory-confirmed influenza infection worldwide. Applying the proportions of children younger than 3 months at time of death to global mortality estimates, we estimated the potential impact of maternal influenza vaccination on in-hospital paediatric-related death.

## 2 Methods

### 2.1 Study design and patients

The FLU GOLD study was initiated in October 2017. We invited our existing global respiratory syncytial virus (RSV) GOLD network [15], consisting of individual investigators, research groups, and clinicians, to share individual-level data of children aged 0–59 months who had died with laboratory-confirmed influenza infection between January 1, 1995 and March 31, 2020. YNL, NIM, FSV, and LJB had full access to all the data in the study. We excluded community deaths due to limited available data (*n* = 8) and children with influenza-related mortality after stem cell transplantation. Additionally, we searched the literature using PubMed for “influenza” combined with “death”, “deaths”, “died”, “mortality”, “fatality”, or “case fatality ratio (CFR)” and “pediatric”, “pediatrics”, “child”, or “children” and invited authors to share additional (unpublished) cases. Collaborators were invited to share data between October 13, 2017, and March 31, 2020 through a link to a questionnaire (**Supplementary Material**) in Research Online, an electronic data capture platform [16].

### 2.2 Definitions

We collected demographic and clinical characteristics and compared these between children from different income groups. Countries of origin were categorised as LMIC (LIC and LMIC combined), upper-middle-income countries (UMIC), and HIC according to the World Bank classifications for 2020 [17]. We compared age distribution at time of death for the 3 income groups and between the following 3 patient populations: children with comorbidities, healthy term children, and healthy preterm children (without comorbidities). A comorbidity was defined as at least one underlying disease, such as congenital heart disease, chronic lung disease or a genetic disorder. Prematurity was defined as gestational age less than 37 weeks. If data for comorbidities or prematurity were not reported, we assumed that the children were healthy term. We calculated weight-for-age z-scores as previously described [15]. We determined the proportion of children who died within the influenza virus epidemic season by comparing age at death and seasonality within the country of origin as estimated by a recent systematic analysis on global patterns of monthly influenza virus activity [18]. We compared the proportion of in-hospital deaths under 6 months of age in our study to the proportions from published studies used for the recent global influenza burden study from the Respiratory Virus Global Epidemiology Network [1].

### 2.3 Age at influenza infection

We calculated age at influenza infection by subtracting the number of days between onset of influenza-related symptoms and influenza-related death from age at influenza-related death. We then determined the proportion of children under 3 months of age at time of influenza infection.

### 2.4 Community-acquired and hospital-acquired influenza-related death

We differentiated between children with community-acquired and hospital-acquired influenza infection. In case the setting where influenza had been acquired was not provided, and if there were no strong indications of nosocomial infection based on timeframe and clinical disease course, we assumed the infection had been community-acquired. We assumed that deaths occurred within the hospital if data on location of death were missing.

### 2.5 Potential impact of maternal influenza vaccination on influenza-related in-hospital death

To evaluate the minimum expected impact of maternal vaccination on influenza-related deaths assuming 100% vaccine efficacy and complete vaccination coverage, we multiplied the proportion of children younger than 3 months at time of community-acquired in-hospital death by the estimated total number of global influenza-associated ALRI in-hospital deaths under 5 years of age for each World Bank income group [1].

### 2.6 Sensitivity analyses

We compared demographic and clinical characteristics between different income groups, excluding cases with missing data for comorbidities or prematurity. We performed subgroup analyses and compared characteristics for children with hospital-acquired and community-acquired influenza-related death. Furthermore, we differentiated between seasonal and pandemic influenza-related deaths by excluding children with influenza A(H1N1)pdm09 who died within the timeframe of the WHO-declared pandemic (June 2009 - August 2010) from the analyses. Lastly, we analysed to what extent our results were sensitive to the contribution of a large number of cases from Ecuador, United Kingdom, Kenya, Turkey and South Africa (*n* = 145) by excluding these countries from our analyses.

### 2.7 Ethical approval

Since de-identified secondary patient data were used in the FLU GOLD study, parental informed consent was waived by the institutional research board of the University Medical Centre Utrecht. Ethical approval was obtained for individual collaborating institutes when required.

### 2.8 Statistical analysis

We report descriptive statistics for all variables. Continuous variables are presented as median with interquartile ranges (IQR). Categorical variables are presented as frequencies and proportions. We used the χ*^2^*-test or the Fisher's exact test to determine statistical significance between groups for categorical parameters. We report conservative exact p values instead of asymptotic p values because of the small sample size. The Mann-Whitney U test was used for all continuous parameters. We applied the Bonferroni correction for multiple testing between World Bank income groups. All statistical analyses were performed with SPSS (version 21·0; IBM Corp, Armonk, NY).

### 2.9 Role of the funding source

The funder had no role in study design, data collection, data analysis, data interpretation, writing of the report, or the decision to submit the paper for publication. YNL, NIM, FSV, and LJB had full access to all the data in the study and NIM had final responsibility for the decision to submit for publication.

## 3 Results

**Fig. 1.**
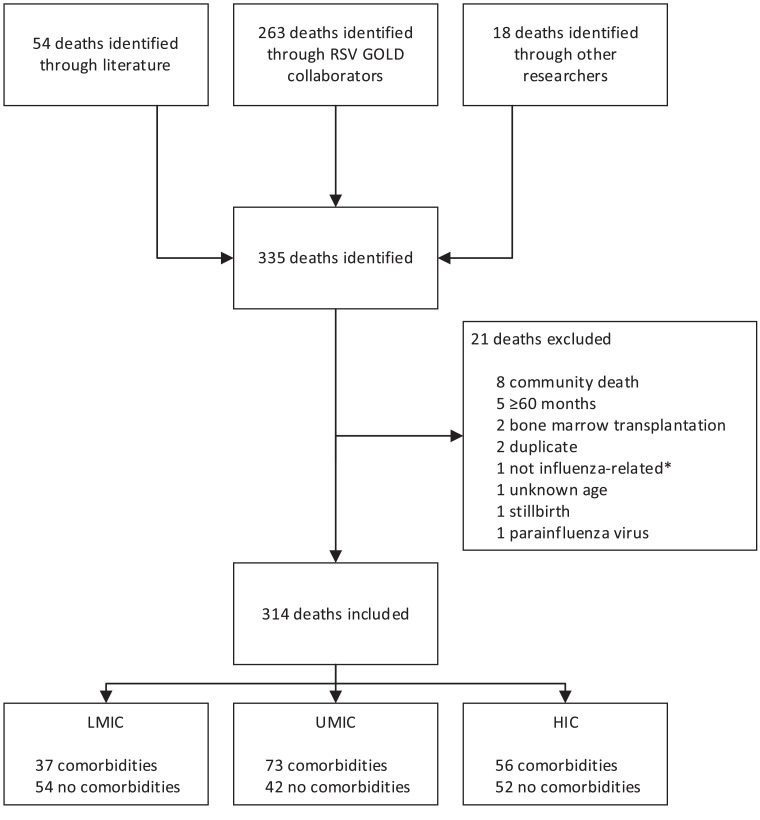
Flowchart of included deaths. RSV=respiratory syncytial virus. GOLD=global online database. LMIC=low-income and lower-middle-income countries. UMIC=upper-middle-income countries. HIC=high-income countries. *For 1 child the collaborator had indicated in the comments that the death was not influenza-related.

**Fig. 2.**
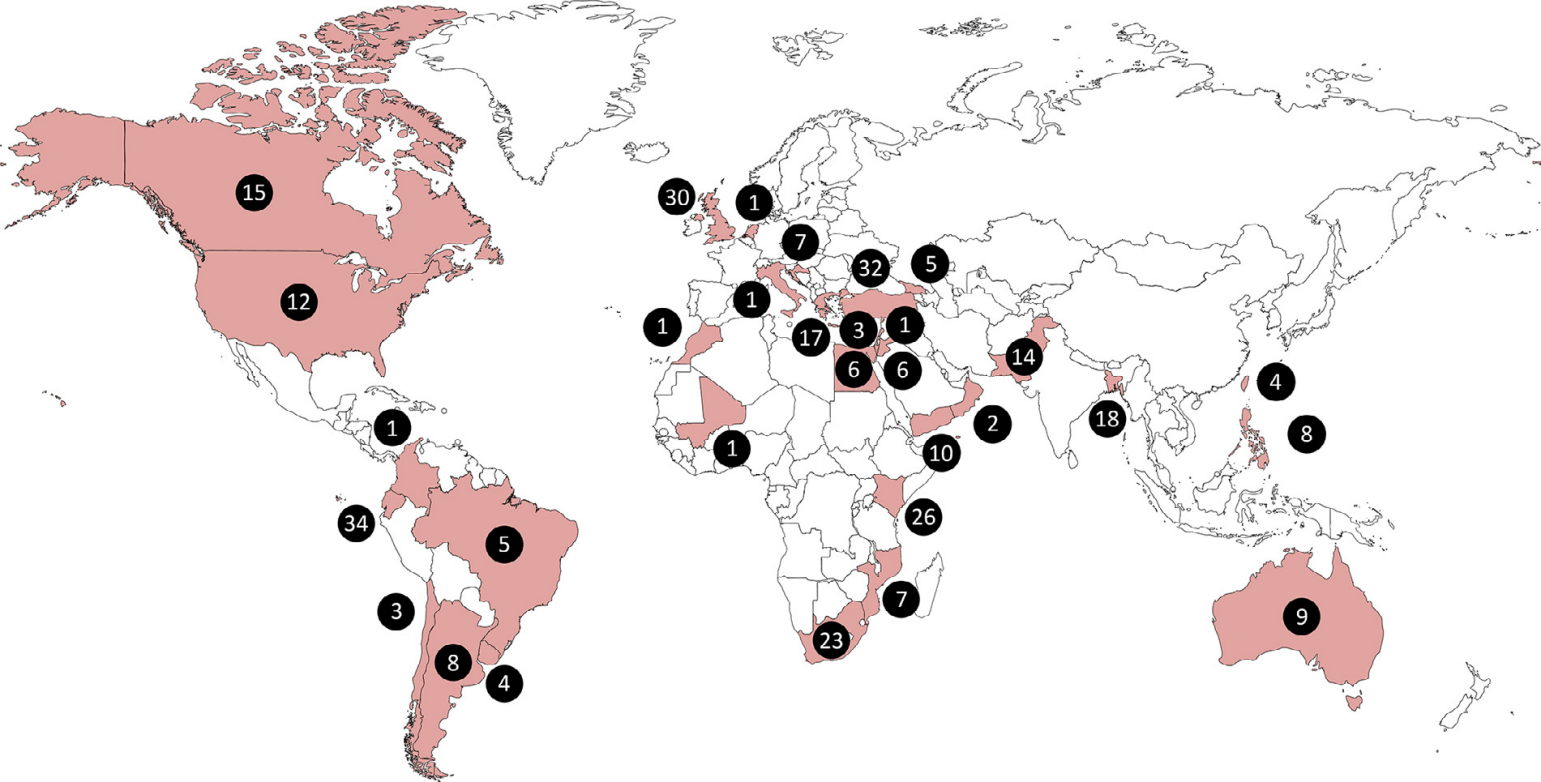
Countries of children with influenza-related in-hospital death included in the analysis. Number of included deaths are given for each country (in pink) from which collaborators shared data.

We obtained data from both published and unpublished cases and included 314 influenza-related deaths from 6 WHO regions and 31 countries (9 LMICs [29%], 9 UMICs [29%], and 13 HICs [42%]) across the world (Figs. 1 and 2, Supplemental Table 1). The majority of cases were shared through the RSV GOLD network and additional cases were identified through the literature search [19,20]. In total, 91 (29·0%) children were from LMICs, 115 (36·6%) were from UMICs and 108 (34·4%) were from HICs.

### 3.1 Clinical and demographic characteristics

Comorbidities were present in at least 166 (52·9%) children and 41 (13·1%) children were born prematurely (Table 1). Paediatric influenza vaccination had been administered to 11 (3·5%) children. Out of 31 children for which data on maternal immunisation were available, 2 children (Chile, *n* = 1 and US, *n* = 1) had mothers who had been vaccinated against influenza during pregnancy. Age at death for these children was 15 and 16 months, respectively. Type of diagnostic influenza tests used and additional characteristics for children from different income countries are included in Supplemental Tables 2 and 3, respectively. Co-pathogens were identified in respiratory samples of 114 children. RSV was the most common co-infection (Supplemental Table 4).

**Table 1:** Clinical and demographic characteristics of children younger than 5 years with influenza-related in-hospital death.

	**LMICs (*n*** **=** **91)**	*p* value*	**UMICs (*n*** **=** **115)**	***p* value†**	**HICs (*n*** **=** **108)**	***p* value‡**	**Total (*n*** **=** **314)**
Male sex	46 (50·5%)	0·58	53 (46·1%)	0·08	62/107 (57·9%)	0·32	161/313 (51·4%)
Age at death (months)	8·6 (4·5–16·6)	0·12	11·5 (4·3–24·0)	0·06	15·5 (7·4–27·0)	<0·001	12·0 (5·0–24·0)
<3 months at death	15 (16·5%)	0·42	14 (12·2%)	0·27	8 (7·4%)	0·07	37 (11·8%)
<6 months at death	30 (33·0%)	0·76	35 (30·4%)	0·07	21 (19·4%)	0·04	86 (27·4%)
Year of death	2015 (2011–2018); *n* = 91	0·32	2014 (2012–2017); *n* = 112	0·004	2012 (2010–2016); *n* = 107	0·001	2013 (2011–2017); *n* = 310
Age at infection (months)	7·6 (2·9–17·4); *n* = 64	0·07	11·3 (3·8–23·9); *n* = 107	0·08	15·9 (6·7–29·7); *n* = 79	0·001	11·5 (4·5–24·0); *n* = 250
<3 months at infection	19 (20·9%)	0·86	22 (19·1%)	0·09	11 (10·2%)	0·05	52 (16·6%)
Type of influenza							
A H1N1 pre-pandemic	3/81 (3·7%)	1·00	5/108 (4·6%)	0·45	2/102 (2·0%)	0·66	10/291 (3·4%)
A H1N1 pdm-09	14/81 (17·3%)	0·03	34/108 (31·5%)	0·09	44/102 (43·1%)	<0·001	92/291 (31·6%)
A H1N2	0/81	1·00	1/108 (0·9%)	1·00	0/102		1/291 (0·3%)
A H3N2	6/81 (7·4%)	0·17	16/108 (14·8%)	0·08	7/102 (6·9%)	1·00	29/291 (10·0%)
B Yamagata	0/81		0/108		0/102		0/291
B Victoria	0/81		0/108	0·49	1/102 (1·0%)	1·00	1/291 (0·3%)
A unsubtyped	32/81 (39·5%)	0·02	25/108 (23·1%)	1·00	23/102 (22·5%)	0·02	80/291 (27·5%)
B unsubtyped	26/81 (32·1%)	0·75	32/108 (29·6%)	0·65	27/102 (26·5%)	0·42	85/291 (29·2%)
Other^	2/81 (2·5%)	0·58	1/108 (0·9%)	1·00	0/102	0·20	3/291 (1·0%)
Comorbidity§	37 (40·7%)	0·001	73 (63·5%)	0·10	56 (51·9%)	0·12	166 (52·9%)
Prematurity§	8 (8·8%)	0·28	16 (13·9%)	0·71	17 (15·7%)	0·20	41 (13·1%)
Gestational age (weeks)	38·0 (34·0–39·0); *n* = 11	0·84	38·0 (35·0–39·0); *n* = 38	0·54	38·0 (34·3–40·0); *n* = 52	0·95	38·0 (35·0–39·0); *n* = 101
Length of stay in hospital (days)	6·5 (3·0–12·8); *n* = 84	0·005	9·0 (4·0–19·0); *n* = 109	0·95	11·5 (3·0–25·8); *n* = 88	0·02	8·0 (3·0–18·0); *n* = 281
Intensive care unit (ICU) admission	47/83 (56·6%)	0·10	75/109 (68·8%)	<0·001	86/89 (96·6%)	<0·001	208/281 (74·0%)
Length ICU admission (days)	4·5 (1·8–12·3); *n* = 38	0·004	10·5 (4·0–24·5); *n* = 52	0·17	8·0 (2·0–22·0); *n* = 83	0·10	8·0 (3·0–19·5); *n* = 173
ICU not available	13/83 (15·7%)	<0·001	0/109		0/89	<0·001	13/281 (4·6%)
Respiratory support	40/82 (48·8%)	<0·001	68/72 (94·4%)	0·17	88/89 (98·9%)	<0·001	196/243 (80·7%)
Mechanical ventilation	16/82 (19·5%)	<0·001	48/72 (66·7%)	<0·001	82/89 (92·1%)	<0·001	146/243 (60·1%)
Respiratory support not available	31/82 (37·8%)	<0·001	0/72		0/89	<0·001	31/243 (12·8%)
Time between onset of symptoms and hospital admission (days)	4·0 (2·0–7·0); *n* = 64	<0·001	2·0 (0·0–4·0); *n* = 110	0·96	2·0 (1·0–3·0); *n* = 80	<0·001	2·0 (1·0–4·3); *n* = 254
Time between onset of symptoms and death (days)	12·0 (7·0–19·8); *n* = 64	0·63	12·0 (7·0–24·0); *n* = 107	0·83	12·0 (5·0–27·0); *n* = 79	0·73	12·0 (6·0–22·3); *n* = 250
Death during influenza season	64 (70·3%)	0·08	65/112 (58·0%)	0·57	60/96 (62·5%)	0·28	189/299 (63·2%)

Data are n (%), n/N (%) or median (IQR); n. Statistical comparisons with χ^2^ test using exact p values, Fisher's exact test or Mann-Whitney U test with p values of less than 0·0167 taken to be significant according to the Bonferroni correction for multiple testing. LMIC=low-income and lower-middle-income countries. UMIC=upper-middle-income countries. HIC=high-income countries. *Low-income or lower-middle-income country versus upper-middle-income country. †Upper-middle-income country versus high-income country. ‡Low-income or lower-middle-income country versus high-income country. ^These children were diagnosed with unsubtyped influenza C. §Considered absent when missing (children with missing data for comorbidity; *n* = 27, and prematurity; *n* = 150).

### 3.2 Age distribution

**Fig. 3:**
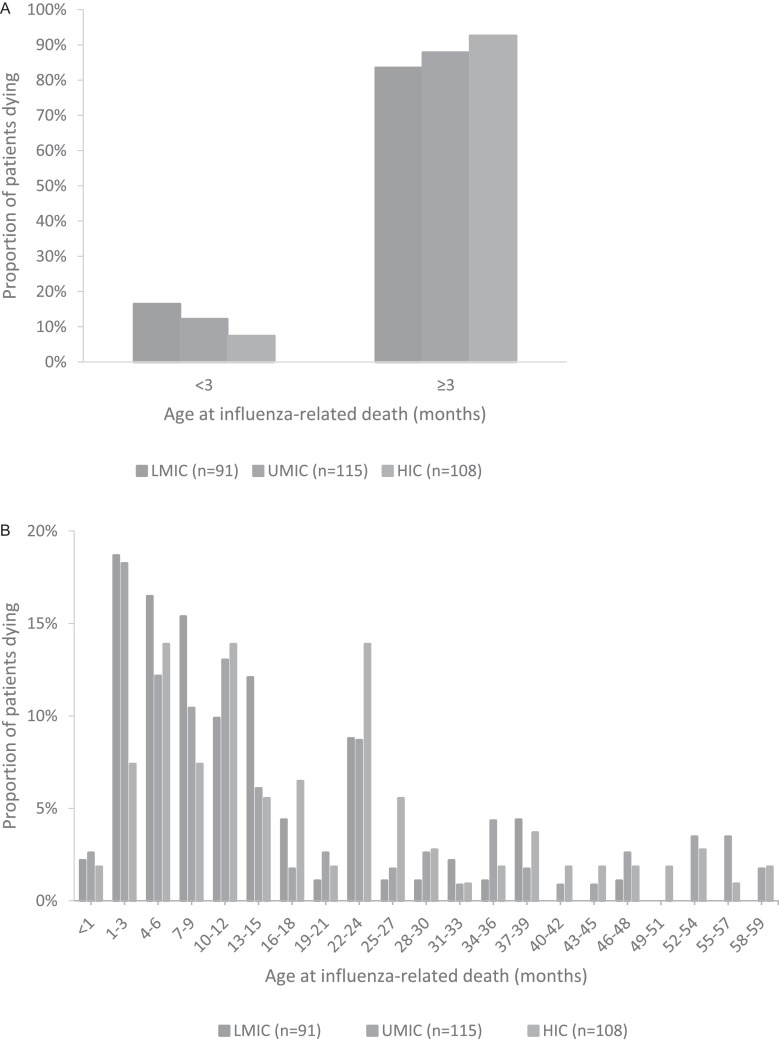
A) Age younger than 3 months or 3 months and older at time of influenza-related in-hospital death for children younger than 5 years from low- and lower-middle-income countries (LMIC), upper-middle-income countries (UMIC), and high-income countries (HIC). B) Age distribution at time of influenza-related in-hospital death for children younger than 5 years from low- and lower-middle-income countries (LMIC), upper-middle-income countries (UMIC), and high-income countries (HIC).

Globally, more than 80% of paediatric influenza-related deaths occurred after the first 3 months of life. The proportion of children younger than 3 months at time of death was 16·5% in LMICs, 12·2% in UMICs and 7·4% in HICs (Fig. 3A). The proportions of children younger than 6 months at time of death were 33·0% in LMICs, 30·4% in UMICs, and 19·4% in HICs. Median age at death was 8·6, 11·5 and 15·5 months for children from LMICs, UMICs, and HICs, respectively (Fig. 3B). Children from LMICs were younger at time of death than children from HICs (*p*<0·001). The age distribution for preterm children without comorbidities, term children with comorbidities, and healthy term children is shown in Supplemental Figure 1.

### 3.3 Hospital-acquired influenza-related death

We separately described variables for 38 (12·1%) children with hospital-acquired influenza-related death and compared these to children with community-acquired influenza-related death (Supplemental Table 5). Age at death, and time between onset of influenza-related symptoms and death did not differ significantly between both groups (*p* = 0·09 and *p* = 0·14, respectively). More children with hospital-acquired influenza-related death were born preterm as compared to children with community-acquired influenza-related death (36·8% versus 9·8%, *p*<0·001). Although timing of intensive care unit (ICU) admission in relation to influenza infection was unknown, all children with hospital-acquired influenza-related death had been admitted to the ICU. This differed from children with community-acquired influenza-related death, of which 70·1% had been admitted to the ICU (*p*<0·001).

### 3.4 Potential impact of maternal vaccination on influenza-related in-hospital death

Based on published global mortality estimates, we calculated that 3339 (UR 1287–9886) influenza-related in-hospital deaths occurred annually in children younger than 3 months (Table 2).

**Table 2:** Global paediatric influenza-associated in-hospital deaths in children younger than 5 years.

	Estimated number of global annual influenza-related ALRI deaths in children younger than 5 years[1]	Proportion of children younger than 3 months (FLU GOLD registry)	Potentially prevented influenza-related ALRI in-hospital death*
LMIC	17,000 (6900–45,100)	17·2%	2924 (1187–7757)
UMIC	3200 (800–14,400)	11·6%	371 (93–1670)
HIC	600 (100–6200)	7·4%	44 (7–459)
Overall			3339 (1287–9886)**

Data are provided in numbers and uncertainty ranges. ALRI=acute lower respiratory infection. LMIC=low-income and lower-middle-income countries. UMIC=upper-middle-income countries. HIC=high-income countries.

*Percentage of children younger than 3 months with in-hospital, community-acquired influenza-related death from the FLU GOLD registry (*n* = 276, Supplemental Table 6) was multiplied with the number of global estimated deaths as reported by Wang et al. [1] for each income group. By analyzing the income groups separately, we accounted for the fact that the proportion of children from LMICs in the FLU GOLD registry is not representative for the total global burden in this population.

**Overall number was calculated by summing up the number of potentially prevented influenza-related ALRI in-hospital deaths for each World Bank income level group.

### 3.5 Sensitivity analyses

When excluding children with hospital-acquired influenza-related mortality (*n* = 38) from the analyses, differences in characteristics between income groups remained unchanged, except for the length of hospital and ICU stay, and the presence of comorbidities between children from LMICs and UMICs (Supplemental Table 6).

We also analyzed our data after excluding children with missing data for prematurity and comorbidities (*n* = 150). Results for age distribution remained unchanged, including a higher age at death for children from HICs (14·5 months [IQR 6·1–28·6]) than from UMICs (12·0 months [IQR 4·0–28·9]) and LMICs (9·2 months [IQR 4·0–23·2]) (Supplemental Table 7), although the difference in age at death for children from HICs compared to children from LMICs was no longer significant. Furthermore, when excluding 26 children with influenza A(H1N1)pdm09 virus infection who died within the timeframe of the WHO-declared pandemic (June 2009 - August 2010), conclusions did not change (Supplemental Table 8).

Lastly, we analysed our data after excluding children from Ecuador, Kenya, United Kingdom, Turkey, and South Africa (*n* = 145), as these accounted for almost half of all influenza-related deaths (Fig. 2). As these were mainly UMIC deaths (*n* = 89), median age at death for children from UMICs increased to 23·5 months (IQR 9·9–39·0) and there were more children with comorbidities from HICs (62·8%) as compared to children from LMICs (30·8%, *p*<0·001) (Supplemental Table 9).

## 4 Discussion

To our knowledge, the FLU GOLD study is the first global case series reporting on influenza-related deaths in children under 5 years of age. We collected demographic and clinical characteristics of children who died with influenza virus infection in the hospital from 31 countries. We showed that more than 80% of children were 3 months or older at time of influenza-related death. This finding implies that maternal vaccine alone would be insufficient to prevent the majority of global paediatric influenza-related in-hospital mortality, given that the duration of protection is limited to the first 3 months of life [12]. We estimated that a total of 3339 influenza-related in-hospital deaths in infants younger than 3 months could have been potentially prevented by maternal vaccination, which is 21·8% of the global influenza-related paediatric mortality burden [1]. Maternal vaccination may have a higher impact on influenza-related paediatric in-hospital deaths in LMICs as we observed a trend towards a higher proportion of children dying below the age of 3 months in LMICs compared to other income regions. Since we reported conservative p values, this difference may be significant in larger sample sizes.

Our findings are in line with a systematic review of the global burden of influenza-associated in-hospital deaths in children under 5 years in which 36% of deaths occurred in children younger than 6 months. Moreover, the highest number of deaths was estimated to occur in children aged 12–59 months, comparable to our results [1].

We found that at least 53% of children had comorbidities which is similar to results from other studies [21], [22] In contrast, in a study from the United Kingdom that evaluated all influenza-related paediatric ICU admissions between 2003 and 2015, nearly four fifths of children had high-risk conditions [23]. The Advisory Committee on Immunization Practices (ACIP) from the United States Centers for Disease Control and Prevention (CDC) recommends vaccination for children aged 6 months and older and recognises that children with the following underlying conditions are at increased risk of developing medical complications attributable to severe influenza infection: chronic pulmonary disease, hemodynamically significant cardiovascular disease, immunosuppression, renal, hepatic, neurological, hematologic, or metabolic disorders [4]. In our registry, congenital heart disease and neurological disease were the most reported comorbidities. Prematurity was present in 13% of children, but data were missing for 48% of all children included in the study. Comorbidities were less often reported in children from LMICs than from UMICs. This may have been caused by lack of available clinical data or limited access to adequate healthcare resources resulting in a higher number of influenza-related deaths in previously healthy children.

Children from LMICs were younger at time of influenza-related death, had a longer time interval between onset of symptoms and hospital admission and were less often admitted to the ICU as compared to children from UMICs and HICs. These findings might be due to poor access to healthcare and limited availability of ICU beds rather than underlying susceptibility of these children to influenza. In contrast, in UMICs and HICs, children may die at an older age due to pre-existing susceptibility from underlying conditions.

Paediatric deaths associated with influenza A(H1N1)pdm09 virus were proportionately higher in HICs than LMICs. This is likely caused by publication bias since there were more studies on influenza A(H1N1)pdm09 virus available from HICs than from LMICs. Furthermore, information on influenza subtype in LMICs was not always available, which also may have contributed to this difference.

Beyond the scope of this paper, we compared our data to 358 previously reported cases from the RSV GOLD I study of children under 5 years of age with RSV-related in-hospital mortality [15], as RSV is currently the most common viral pathogen causing ALRI in young children. Children with influenza-related death were substantially older than children with RSV-related death (Supplemental Table 10, Supplemental Figure 2). This difference was also demonstrated in other studies [24]. In a prospective Dutch birth cohort of 4072 premature infants [25], 181 infants were hospitalised with RSV infection and 2 with influenza infection in the first year of life (data not shown), indicating that severe ALRI due to influenza is less prevalent than RSV in younger children. Reasons for this age difference are likely multifactorial. In contrast to influenza, RSV predominantly affects the airways and causes greater epithelium damage [26], leading to significant airway obstruction and fatality in infants who have smaller airways than older children. Additionally, coinfections, which could cause mortality also in older children, may occur less often in children with RSV infection compared to children with influenza infection [27].

For the first time, we report global age-stratified data for children dying with influenza. This enabled us to assess the potential impact of maternal vaccination on paediatric influenza-related mortality. Furthermore, we verified each case through direct communication with the local collaborators.

There are also limitations to this study. First, our results reflect only a small proportion of paediatric influenza-related mortality occurring worldwide, since limited data are available due to lack of diagnostic testing, in particular from children who died outside of the hospital. Our database included only 18 (5·7%) children from LICs, and this proportion should be much higher based on global mortality burden estimates. Since children dying in the community may be younger and are mainly from LMICs, our results are an underestimation of the potential impact of maternal vaccination on paediatric influenza-related mortality in LMICs. Furthermore, since we only included individual-level patient data, it was not possible to use larger published case series from the US for which only aggregated data could be provided [3,20,[28], [29], [30], [31]]. The percentage of children younger than 6 months within the under-five age group in these case series ranged between 19% and 35%, which is slightly higher than we report for HICs (19%). Second, we were unable to assess the potential impact of maternal vaccination on stillbirths associated with in utero influenza exposure. Furthermore, we were unable to assess the indirect impact of maternal vaccination on paediatric mortality due to bacterial pneumonia following influenza infection, all-cause ALRI, deaths prevented due to herd immunity including young siblings, or impact on postpartum maternal death and subsequent effects on the health of the child. This limitation resulted in an underestimation of the potential impact of maternal influenza vaccination on paediatric mortality. Third, we did not account for maternal vaccination coverage, vaccine efficacy and potential programmatic limitations. Data on maternal immunization during pregnancy was available for only 31 patients. For this reason we may show an overestimation of vaccine impact. Fourth, our literature search terms were more limited than those used for the global influenza burden study [1] resulting in missed published deaths (91 deaths from 18 studies). From the missed published deaths, age distribution data were available for 15 children, of which 3 (20%) were younger than 6 months at the time of influenza-related death, which is similar to what we observed (27%). We therefore expect that this limitation does not have a major impact on our study. Data were unavailable on the proportion of children younger than 3 months in the missed deaths. Fifth, we have based our estimations on the global influenza estimates from the global burden study, which has its own limitations such as heterogeneity of studies, several forms of bias and scarcity of data, which could have caused both overestimations as underestimations of paediatric influenza-related death burden according to the authors and therefore could lead to over- or underestimation of vaccine impact on mortality [1]. Moreover, data from LMICs were limited in this study which likely leads to an underestimation of mortality burden. Sixth, some authors did not respond to our invitation to collaborate, which could have led to non-response bias. Seventh, data on location of death were not always available. We assumed that these deaths occurred within the hospital due to the limited number of studies and lack of available influenza testing within the community. Furthermore, the majority of children with missing data for location of death were from HICs. It is unlikely that these children would not have been admitted to hospital. Finally, data for prematurity and comorbidities were often missing, in particular from LMICs, which could have caused an underestimation of the number of children with prematurity and comorbidity. However, when we excluded children with missing data in the sensitivity analyses, the main results from this study remained unchanged.

There are important factors to support maternal influenza vaccination: it has proved to be safe [11] and effective in preventing influenza-associated ALRI in both infants and pregnant women, the latter forming a substantial risk group for influenza-associated hospitalisation [32] and death [33]. Furthermore, maternal influenza vaccination decreased all-cause ALRI hospitalisations in infants during the first 3 months of life [34]. A study on maternal influenza vaccination among HIV-infected and HIV-uninfected pregnant women in South Africa showed that antenatal influenza vaccination campaigns in South Africa would be cost-effective [35]. However, we showed that the impact on paediatric influenza-related death may be limited since more than 80% of global paediatric influenza-related in-hospital deaths occurred after the first 3 months of life, which is beyond the timeframe in which maternally-derived anti-influenza antibodies are expected to have protective effect. Although maternal influenza vaccination may have more impact in LMICs where children with influenza infection die at a younger age, additional immunisation strategies and more high-quality patient data from LMICs are required to prevent global paediatric influenza-related death. Studies to develop a safe and effective influenza vaccine for children younger than 6 months could be considered. In the context of SDG 3·1 and 3·2, maternal vaccination should continue to be emphasized and additional strategies are needed to target influenza-related under-five mortality. The missing data, global coverage, and data quality in this study should be taken into consideration for further interpretation of the results.

*FLU GOLD collaborators are:

Kentigern Thorburn^1^, Marta Nunes^2^, Richard Chawana^2^, Shabir A. Madhi^2^, Anna C. Vittuci^3^, Quique Bassat^4,5,6,7,8^, Azucena Bardají^4,5,8^, Edward Goka^9^, Srđan Roglić^10^"?>, Bosco Paes^11^, LouAnn Elliott^11^, Hitoshi Oshitani^12^, Socorro Lupisan^13^, Angela Gentile^14^, María Florencia Lucion^14^, Michael C. Spaeder^15^, Warwick Butt^16^, Jenny Thompson^16^, Asuncion Mejias^17^, Octavio Ramilo^17^, Rodrigo A. Fasce^18^, Marta Werner^19^, Diego R. Hijano^20^, Kim J. Allison^20^, Márcia Rosane Pires^21^, Fernanda de-Paris^21^, Giorgi Chakhunashvili^22^, Irakli Karseladze^23^, Grieven P. Otieno^24^, D. James Nokes^24^, Oded Scheuerman^25^, Dario Prais^25^, Mohammed Al Amad^26^, Abdul Wahed Al Serouri^26^, Asad Ali^27^, Mohammad Tahir Yousafzai^27^, Alfredo Bruno^28,29^, Domenica de Mora^28^, Jenny Ojeda^30^, Ghassan Dbaibo^31^, Rima Hanna-Wakim^31^, Vassiliki Papaevangelou^32^, Elpiniki Kartisouni^32^, Cheryl Cohen^33^, Sibongile Walaza^33^, Rosalie S. Linssen^34^, Hsin Chi^35^, Aykut Eşki^36^, Esen Demir^37^, Senjuti Saha^38^, Samir K Saha^38^, Anthony A. Sochet^39^, Beatriz E. Teppa-Sanchez^39^, Thyyar M. Ravindranath^40^, J. Scott Baird^40^, Shaun K. Morris^41^, Waison Wong^41^, Robert F. Breiman^42^, Emily S. Gurley^43^, Shams El Arifeen^44^, Nega Assefa^45^, J. Anthony G. Scott^46^, Dickens Onyango^47^, Karen L. Kotloff^48^, Samba O. Sow^49^, Inacio Mandomando^50,51^, Ikechukwu U. Ogbuanu^52^, Amara Jambai^53^, Tanil Kendirli^54^, Edin Botan^54^, Franco Díaz Rubio^55^, Alberto Serra^56^, Luis Martinez^57^, Luis Pedroso^58^, Soledad Menta^59^, Rosalba Pardo^60^, Alejandro Donoso^61^, Syed Faisal Mahmood^62^, Naveera Khan^63^

1 Alder Hey Children's Hospital, and The University of Liverpool, Liverpool, UK
2 South African Medical Research Council: Vaccines and Infectious Diseases Analytics Research Unit, School of Pathology, Faculty of Health Sciences, University of the Witwatersrand; and Department of Science and Technology/National Research Foundation: Vaccine Preventable Diseases Unit, University of the Witwatersrand, Johannesburg, South Africa
3 Bambino Gesù Hospital, Rome, Italy
4 ISGlobal, Hospital Clínic - Universitat de Barcelona, Barcelona, Spain
5 Centro de Investigação em Saúde de Manhiça (CISM), Maputo, Mozambique
6 ICREA, Pg. Lluís Companys 23, 08,010 Barcelona, Spain
7 Paediatrics Department, Hospital Sant Joan de Déu, Universitat de Barcelona, Esplugues, Barcelona, Spain
8 Consorcio de Investigación Biomédica en Red de Epidemiología y Salud Pública (CIBERESP), Madrid, Spain
9 Institute of Inflammation and Repair, The University of Manchester, Manchester, UK
10 University Hospital for Infectious Diseases, Zagreb, Croatia
11 Department of Pediatrics (Neonatal Division), McMaster University, Hamilton, Canada
12 Department of Virology, Tohoku University Graduate School of Medicine, Sendai, Japan
13 Research Institute for Tropical Medicine, Muntinlupa City, Philippines
14 Department of Epidemiology, Ricardo Gutiérrez Children's Hospital, Buenos Aires, Argentina
15 University of Virginia School of Medicine, Charlottesville, USA
16 Department of Paediatric Intensive Care, Royal Children's Hospital, Melbourne, Australia
17 Department of Paediatrics, Division of Infectious Diseases, and Center for Vaccines and Immunity, Nationwide Children's Hospital, Ohio State University, Columbus, USA
18 Department of Viral Diseases, Public Health Institute of Chile, Santiago, Chile
19 Epidemiology Unit Hospital Guillermo Grant Benavente, Concepcion, Chile
20 Paediatric Infectious Diseases, St. Jude Children's Research Hospital, Memphis, USA
21 Hospital de Clínicas de Porto Alegre, Universidade Federal do Rio Grande do Sul, Porto Alegre, Brazil
22 One Health Division, National Center for Disease Control and Public Health, Tbilisi, Georgia
23 Vaccine Preventable and Respiratory Disease Division, National Center for Disease Control and Public Health, Tbilisi, Georgia
24 Kenya Medical Research Institute, Wellcome Trust Research Programme, Centre for Geographic Medicine Research - Coast, Kilifi, Kenya
25 Schneider Children's Medical Center of Israel, Petah Tikva, Sackler school of medicine, Tel Aviv university, Tel Aviv, Israel
26 Yemen Field Epidemiology Training Program (Yemen-FETP), Sana'a, Yemen
27 Department of Paediatrics and Child Health, Aga Khan University Hospital, Karachi, Pakistan
28 Instituto Nacional de Investigación en Salud Publica, Guayaquil, Ecuador
29 Universidad Agraria del Ecuador, Guayaquil, Ecuador
30 Ministerio de Salud Pública del Ecuador, Quito, Ecuador
31 American University of Beirut Medical Center, Beirut, Lebanon
32 Third Department of Paediatrics, National and Kapodistrian University of Athens, Athens, Greece
33 National Institute for Communicable Diseases (NICD), Johannesburg, South Africa
34 Paediatric Intensive Care Unit, Emma Children's Hospital Amsterdam UMC, Amsterdam, the Netherlands
35 Department of Paediatric Infectious Disease, MacKay Children's Hospital, Taipei, Taiwan
36 University of Health Sciences, Department of Pediatric Pulmonology, Dr. Gazi Yaşargil Women's and Children's Health, Education and Research Hospital, Diyarbakır, Turkey
37 Department of Paediatric Pulmonology, Ege University Medical Faculty, Ege University Children's Hospital, Izmir, Turkey
38 Child Health Research Foundation (CHRF), Dhaka, Bangladesh
39 Division of Paediatric Critical Care Medicine, Johns Hopkins All Children's Hospital, St Petersburg, USA
40 Division of Paediatric Critical Care Medicine, Morgan Stanley Children's Hospital of NewYork-Presbyterian, New York, New York, USA
41 Division of Infectious Diseases, The Hospital for Sick Children, Toronto, Canada
42 Emory University, Global Health Institute, Child Health and Mortality Prevention Surveillance (CHAMPS) network, Atlanta, USA
43 Johns Hopkins Bloomberg School of Public Health, Johns Hopkins University, Baltimore, Maryland, USA and International Centre for Diarrhoeal Disease Research, Dhaka, Bangladesh
44 International Centre for Diarrhoeal Disease Research, Dhaka, Bangladesh
45 College of Health and Medical Sciences, Haramaya University, Harar, Ethiopia
46 London School of Hygiene & Tropical Medicine, London, United Kingdom
47 Kisumu County Department of Health, Kisumu, Kenya
48 Department of Pediatrics, Center for Vaccine Development and Global Health and Division of Infectious Disease and Tropical Pediatrics, University of Maryland School of Medicine, Baltimore, Maryland, USA
49 Center for Vaccine Development, Bamako, Mali
50 Centro de Investigação em Saúde de Manhiça (CISM), Maputo, Mozambique
51 Instituto Nacional de Saúde (INS), Ministério da Saúde, Maputo, Mozambique
52 Crown Agents Sierra Leone, Freetown, Sierra Leone
53 Ministry of Health and Sanitation, Freetown, Sierra Leone
54 Division of Paediatric Critical Care, Ankara University School of Medicine, Ankara, Turkey
55 Instituto de Ciencias e Innovación en Medicina, Universidad del Desarrollo, Santiago, Chile
56 Casa de Galicia, Montevideo, Uruguay
57 Hospital Paysandu, Paysandu, Uruguay
58 Hospital Regional Salto, ASSE, Salto, Uruguay
59 Hospital Tacuarembo, Tacuarembo, Uruguay
60 Clínicia Infantil de Colsubsidio, Bogotá, Colombia
61 Hospital La Florida, Santiago, Chile
62 Section of Infectious Diseases, Department of Medicine, Aga Khan University, Karachi, Pakistan
63 Research Facilitation Office, Medical College, Aga Khan University.

## 5 Data sharing statement

The FLU GOLD team believes data sharing is important for the global scientific community. Our data are available upon request to the corresponding author, except for personally identifiable data due to privacy reasons.

## 6 Authors’ contributions

NIM, IR, LJB, HN and MN conceptualized the study. IR and FSV collected the data. YNL and FSV Analyzed the data. NIM, YNL and LJB interpreted the results. YNL wrote the initial draft. All authors critically reviewed and revised the manuscript. YNL, FSV, NIM and LJB had full access to all the data in the study and the corresponding author had final responsibility for the decision to submit for publication.

## 7 Funding

This publication is based on research funded in part by the Bill & Melinda Gates Foundation. The findings and conclusions contained within are those of the authors and do not necessarily reflect positions or policies of the Bill & Melinda Gates Foundation.

## Declaration of Competing Interest

LJB reports grants from Bill and Melinda Gates Foundation, during the conduct of the study. LJB has regular interaction with pharmaceutical and other industrial partners. He has not received personal fees or other personal benefits. The University Medical Centre Utrecht (UMCU) has received major funding obtained by LJB (>€100,000 per industrial partner) for investigator initiated studies from AbbVie, MedImmune, Janssen, the Bill and Melinda Gates Foundation, Nutricia (Danone) and MeMed Diagnostics. UMCU has received major cash or in kind funding as part of the public private partnership IMI-funded RESCEU project from GSK, Novavax, Janssen, AstraZeneca, Pfizer and Sanofi. UMCU has received major funding by Julius Clinical for participating in the INFORM study sponsored by MedImmune. UMCU has received minor funding for participation in trials by Regeneron and Janssen from 2015 to 2017 (total annual estimate less than €20,000). UMCU received minor funding for consultation and invited lectures by AbbVie, MedImmune, Ablynx, Bavaria Nordic, MabXience, Novavax, Pfizer, Janssen (total annual estimate less than €20,000). LJB is the founding chairman of the ReSViNET Foundation. HN reports grants and personal fees from the World Health Organisation, personal fees from the Bill & Melinda Gates Foundation, personal fees from Sanofi, outside the submitted work. MCN reports grants from the Bill & Melinda Gates Foundation, personal fees from Sanofi Pasteur, personal fees from Pfizer, outside the submitted work. NIM has regular interaction with pharmaceutical and other industrial partners. She has not received personal fees or other personal benefits. UMCU received minor funding for NIMs consultation and invited lectures by Abbvie and Merck. All other authors have nothing to declare.

## Appendix Supplementary materials

Image, application 1
