# Supplementary material for: Estimated impact of maternal vaccination on global paediatric influenza-related in-hospital mortality: A retrospective case series
Source: eClinicalMedicine. 2021 Jun 10;37:100945. doi: 10.1016/j.eclinm.2021.100945 (PMC8343247; doi:10.1016/j.eclinm.2021.100945)
Supplement: Supplementary file 1 [file mmc1.docx]

**SUPPLEMENTARY MATERIAL**

**Supplemental FLU GOLD questionnaire**

Inclusion criteria: Influenza confirmed illness and death in children under 5 years, after January 1 1995.

Exclusion criteria: Stem cell transplantation or bone marrow transplantation

Contact details

Title …

First name …

Last name …

Email address …

Telephone number …

Country …

Profession …

Institute affiliated …

Institute where Influenza mortality case is collected

*(please fill in all fields if information is not available write N/A)*

Name of institute …

City/Town …

Region/Province/State …

Country …

Influenza diagnosis

1.1 Diagnostic test used (several answers possible)

- PCR
- immunofluorescence
- enzyme immuno assay
- culture
- serology
- other, specify: …

1.2 Type of influenza

- Influenza A H1N1 pre-pandemic
- Influenza A H1N1 pdm09
- Influenza A H1N2
- Influenza A H3N2
- Influenza B Yagamata
- Influenza B Victoria
- Influenza A unsubtyped
- Influenza b unsubtyped
- no data available
- other, specify: …

Age Patient

2.1 Age at moment of death (days is preferred over months, if available)

…. measured in days

…. measured in months

2.2 Date of death: month/year (jan-dec)/(1995-...)

Basic patient characteristics

3.1 Gender

- male
- female
- no data available

3.2 Severe underlying disease/comorbidity (if yes, several answers possible)

yes/no/ no data available

- congenital heart disease
- chronic lung disease
- primary/congenital immunodeficiency (not HIV)
- genetic/chromosomal disease
- Down syndrome
- neuromuscular disease
- neurodevelopmental disease
- airway abnormality
- malignancy
- malaria
- HIV/AIDS if yes, CD4 count …
- TB
- other, specify: …

3.3 Length/height at admission

…. measured in centimeters (cm)

…. measured in inches (in)

no data available

3.4 Weight at admission

- - - - …. measured in kilogram (kg)
      - …. measured in pounds (lbs)
      - no data available

3.5 Current type of feeding
*Current feeding if child is < 4 months of age. If child is > 4 months of age report feeding in first 4 months of life. Exclusive breast feeding defined as: no other food or drink, not even water, except breast milk (including milk expressed or from a wet nurse) for 4 months, but allows the infant to receive ORS, drops and syrups (vitamins, minerals and medicines)*

- exclusive breast feeding
- exclusive bottle feeding
- mixed breast & bottle feeding
- no data available
- other, specify: …

Perinatal history

4.1 Prematurity yes/no/ no data available
*< 37 completed weeks of gestation*

4.2 Gestational age … measured in weeks / no data available

estimated by: LMP/ultrasonography/Fundal height/ballard score/unknown

4.3 Birth weight

….. measured in kilograms

….. measured in pounds

no data available

4.4 Mother immunized during pregnancy yes/no/ no data available

Hospital admission

5.1 Hospitalisation yes/no/ no data available

5.2 Length of stay in hospital … measured in days/ no data available

5.3 Intensive care admission yes/no/no ICU available/ no data available

if yes … days

5.4 Respiratory support yes/no/no respiratory support available/ no data available

if yes … days

If yes, type of respiratory support:

- Mechanical ventilation
- Non-invasive ventilatory support (CPAP or high flow nasal canula)
- Oxygen

5.5 Time interval between onset of Influenza related symptoms and first contact with health care provider … measured in days / no data available

5.6 Time interval between onset of influenza related symptoms and hospital admission
 … measured in days/no data available

5.7 Did patient die in hospital? yes/no/ no data available

5.8 Time interval between onset of Influenza related symptoms and death
 … measured in days/ no data available

5.9 Time interval between hospital admission and death
 …measured in days/no data available

5.10 Hospital-acquired influenza infection yes/no/ no data available

Clinical characteristics.

6.1 Clinical symptoms at diagnosis (several answers possible):

- cough
- difficult breathing
- fast breathing (age < 2 months, ≥ 60 breaths/min; age 2–11 months, ≥ 50/min; age 1–5 years, ≥ 40/min)
- chest indrawing
- central cyanosis
- stridor
- inability to breastfeed or drink, vomiting everything
- lethargy, reduced level of consciousness or convulsions
- fever (temperature >38°C/100°F)
- no data available

6.2 Oxygen saturation (SpO2) upon hospital admission

yes, percentage…%/ no data available

6.3 WBC count at diagnosis

- count…..cu mm
- no data available

6.4 Haemoglobin level at diagnosis

- measured in g/dl……. g/dL
- measured in mmol/l…… mmol/l
- no data available

6.5 Were respiratory samples tested for other pathogens?

- yes
- no
- no data available

if yes, by (several answers possible):

- PCR
- immunofluorescence
- enzyme immuno assay
- culture
- no data available

6.6 Other respiratory virus or bacteria present in respiratory sample

yes/no/no data available

if yes (several answers possible):

- RSV
- Para-influenza virus
- Human metapneumovirus
- Adenovirus
- Rhinovirus
- Streptococcus pneumoniae
- Haemophilus influenzae
- Mycoplasma pneumonia
- Enterovirus
- Bocavirus
- Coronavirus
- Pertussis
- Other, specify….

6.7 Micro-organisms present in other samples

yes/no/ no data available

If yes:
 Sample

- blood micro-organisms found: ….
- CSF micro-organisms found: ….
- urine micro-organisms found: ….
- other, specify (both sample type and found micro-organisms)…….

6.8 Were there other clinical diagnoses for this hospital admission?

yes/no/ no data available

If yes, clinical diagnosis (several answers possible):

- Meningitis
- Encephalopathy
- Sepsis
- Pneumonia/LRTI
- Other, specify….

6.9 Cause of death …./no data available

Treatment and prophylaxis

7.1 Specific Influenza treatment yes/no/no data available

If yes:

- neuraminidase inhibitors (oseltamivir (tamiflu), zanamivir, laminamivir)
- M2 inhibitors (amantadine, rimantadine)
- inhaled bronchodilators
- systemic corticosteroids
- ribavirin
- antibiotics
- chest physiotherapy
- maintaining hydration and fluid balance
- epinephrine
- nebulized hypertonic saline
- normal saline
- antitussive, expectorants, decongestants
- other, specify….

7.2 Immunisation status

- received vaccines
- not vaccinated
- no data available

Which vaccines were received (multiple answers possible)

- BCG at age ….
- Hepatitis B at age ….
- Polio at age ….
- DTP containing vaccine at age ….
- Haemophilus influenzae type b at age ….
- Pneumococcal at age ….
- Rotavirus at age ….
- Measles at age ….
- Rubella at age ….

7.3 Influenza immunisation status

- received vaccinations 1 dose/2 doses
- not vaccinated
- no data available

7.4 Who is routinely vaccinated against influenza (as part of the National Health Program)

- pregnant women
- children
- elderly
- healthcare workers
- people with co-morbidity
- no data available

Sociodemographic characteristics

8.1 Other children (< 5 years old) in household

yes/no/ no data available

8.2 Day care attendance or shared care where other children are present

yes/no/ no data available

8.3 Maternal education (highest level attended)

- uneducated
- primary school level
- secondary school level
- university level
- no data available

8.4 Paternal education (highest level attended)

- uneducated
- primary school level
- secondary school level
- university level
- no data available

8.5 Paternal smoking yes/no/no data available

8.6 Maternal smoking yes/no/no data available

8.7 Comments or additional information

***Supplemental Table 1*: Origin of data for children younger than 5 years with influenza-related in-hospital death**

| **Country** | **Children with comorbidity (n=166)** | **Healthy term children  (n=140)** | **Healthy preterm children (n=8)** |
| --- | --- | --- | --- |
| Low-income |  |  |  |
| Mali | 0 | 1 (0·7%) | 0 |
| Mozambique | 3 (1·8%) | 4 (2·9%) | 0 |
| Yemen | 0 | 10 (7·1%) | 0 |
| Lower middle-income |  |  |  |
| Bangladesh | 10 (6·0%) | 8 (5·7%) | 0 |
| Egypt | 0 | 6 (4·3%) | 0 |
| Kenya | 17 (10·2%) | 8 (5·7%) | 1 (12·5%) |
| Morocco | 0 | 1 (0·7%) | 0 |
| Pakistan | 7 (4·2%) | 4 (2·9%) | 3 (37·5%) |
| Philippines | 0 | 8 (5·7%) | 0 |
| Upper middle-income |  |  |  |
| Argentina | 5 (3·0%) | 2 (1·4%) | 1 (12·5%) |
| Brazil | 4 (2·4%) | 1 (0·7%) | 0 |
| Colombia | 1 (0·6%) | 0 | 0 |
| Ecuador | 16 (9·6%) | 18 (12·9%) | 0 |
| Georgia | 3 (1·8%) | 2 (1·4%) | 0 |
| Jordan | 0 | 6 (4·3%) | 0 |
| Lebanon | 1 (0·6%) | 0 | 0 |
| South Africa | 15 (9·0%) | 8 (5·7%) | 0 |
| Turkey | 28 (16·9%) | 3 (2·1%) | 1 (12·5%) |
| High-income |  |  |  |
| Australia | 6 (3·6%) | 3 (2·1%) | 0 |
| Canada | 12 (7·2%) | 3 (2·1%) | 0 |
| Chile | 3 (1·8%) | 0 | 0 |
| Croatia | 5 (3·0%) | 2 (1·4%) | 0 |
| Greece | 11 (6·6%) | 6 (4·3%) | 0 |
| Israel | 1 (0·6%) | 1 (0·7%) | 1 (12·5%) |
| Italy | 1 (0·6%) | 0 | 0 |
| Netherlands | 1 (0·6%) | 0 | 0 |
| Oman | 0 | 2 (1·4%) | 0 |
| Taiwan | 3 (1·8%) | 1 (0·7%) | 0 |
| United Kingdom | 7 (4·2%) | 22 (15·7%) | 1 (12·5%) |
| United States of America | 6 (3·6%) | 6 (4·3%) | 0 |
| Uruguay | 0 | 4 (2·9%) | 0 |

Data are n (%).

***Supplemental Table 2*: Influenza diagnostic tests used per income region**

| **Influenza diagnostic test used** | **LIC (n=18)** | **LMIC (n=73)** | **UMIC (n=115)** | **HIC (n=107)** |
| --- | --- | --- | --- | --- |
| PCR | 18 (100%) | 73 (100%) | 101 (87·8%) | 81 (75·7%) |
| Immunofluorescence | 0 | 0 | 23 (20·0%) | 19 (17·8%) |
| Enzyme immunoassay | 0 | 0 | 1 (0·9%) | 11 (10·3%) |
| Culture | 0 | 18 (24·7%) | 6 (5·2%) | 7 (6·5%) |
| Serology | 0 | 17 (23·3%) | 0 | 0 |
| Other | 0 | 0 | 0 | 1 (0·9%) |

LIC=low-income countries. LMIC=lower middle-income countries. UMIC=upper middle-income countries. HIC=high-income countries. Data are n (%). Data were missing for 1 child from a high-income country that was transferred from outside the hospital without documentation. One child from a lower middle-income country received 2 different diagnostic tests (PCR and culture) and 17 children received 3 different diagnostic tests (PCR, culture and serology). Six children from upper middle-income countries received 2 different diagnostic tests (PCR and immunofluorescence; PCR and culture) and 5 children received 3 different diagnostic tests (PCR, immunofluorescence and culture). Ten children from high-income countries received 2 different diagnostic tests (PCR and immunofluorescence; PCR and enzyme immunoassay; immunofluorescence and culture; enzyme immunoassay and culture; PCR and another diagnostic test) and 1 child received 3 different diagnostic tests (immunofluorescence, enzyme immunoassay and culture).

***Supplemental Table 3*: Additional characteristics of children younger than 5 years with influenza-related in-hospital death**

|  | **LMICs (n=91)** | **p value*** | **UMICs (n=115)** | **p value†** | **HICs (n=108)** | **p value‡** | **Total (n=314)** |
| --- | --- | --- | --- | --- | --- | --- | --- |
| Comorbidity§ | 37 (40·7%) | 0·001 | 73 (63·5%) | 0·10 | 56 (51·9%) | 0·12 | 166 (52·9%) |
| Congenital heart disease | 15 (16·5%) | 1·00 | 18 (15·7%) | 0·18 | 25 (23·1%) | 0·29 | 58 (18·5%) |
| Chronic lung disease | 1 (1·1%) | <0·001 | 21 (18·3%) | 0·19 | 12 (11·1%) | 0·004 | 34 (10·8%) |
| Immune disorder | 5 (5·5%) | 0·22 | 12 (10·4%) | 0·003 | 1 (0·9%) | 0·10 | 18 (5·7%) |
| Genetic/chromosomal disease | 6 (6·6%) | 1·00 | 7 (6·1%) | <0·001 | 26 (24·1%) | 0·001 | 39 (12·4%) |
| Down syndrome | 3 (3·3%) | 1·00 | 3 (2·6%) | 0·49 | 5 (4·6%) | 0·73 | 11 (3·5%) |
| Neurological disease | 5 (5·5%) | 0·03 | 18 (15·7%) | 0·30 | 23 (21·3%) | 0·002 | 46 (14·6%) |
| Airway abnormality | 2 (2·2%) | 0·30 | 7 (6·1%) | 1·00 | 6 (5·6%) | 0·29 | 15 (4·8%) |
| Malaria | 1 (1·1%) | 0·44 | 0 |  | 0 | 0·46 | 1 (0·3%) |
| HIV/AIDS | 9 (9·9%) | 0·43 | 7 (6·1%) | 0·01 | 0 | 0·001 | 16 (5·1%) |
| Tuberculosis | 0 | 0·13 | 4 (3·5%) | 0·12 | 0 |  | 4 (1·3%) |
| Malignancy | 0 | 0·01 | 8 (7·0%) | 0·57 | 5 (4·6%) | 0·06 | 13 (4·1%) |
| Other comorbidity | 6 (6·6%) | 0·79 | 9 (7·8%) | 0·41 | 5 (4·6%) | 0·76 | 20 (6·4%) |
| Biliary atresia | 0 | 1·00 | 1 (0·9%) | 1·00 | 1 (0·9%) | 1·00 | 2 (0·6%) |
| Liver disease | 0 | 1·00 | 1 (0·9%) | 1·00 | 0 |  | 1 (0·3%) |
| Renal disease | 4 (4·4%) | 0·70 | 3 (2·6%) | 0·25 | 0 | 0·04 | 7 (2·2%) |
| Congenital abnormality | 1 (1·1%) | 1·00 | 1 (0·9%) | 1·00 | 0 | 0·46 | 2 (0·6%) |
| Metabolic disorder | 0 |  | 0 | 0·23 | 2 (1·9%) | 0·50 | 2 (0·6%) |
| Endocrine disorder | 0 | 0·50 | 2 (1·7%) | 0·50 | 0 |  | 2 (0·6%) |
| Pulmonary hypertension | 0 |  | 0 | 0·48 | 1 (0·9%) | 1·00 | 1 (0·3%) |
| Shortgut syndrome | 0 |  | 0 | 0·48 | 1 (0·9%) | 1·00 | 1 (0·3%) |
| Developmental disorder | 1 (1·1%) | 1·00 | 1 (0·9%) | 1·00 | 0 | 0·46 | 2 (0·6%) |
| Birth weight (kg) | 2·6 (2·3-3·3); n=13 | 0·86 | 2·7 (2·2-3·4); n=38 | 0·88 | 3·0 (2·2-3·3); n=49 | 0·97 | 2·7 (2·3-3·3); n=100 |
| Mother immunised during pregnancy | 0/4 |  | 0/8 | 1·00 | 2/19 (10·5%) | 1·00 | 2/31 (6·5%) |
| Exclusive breast feeding <3 months | 15/17 (88·2%) | 0·001 | 12/32 (37·5%) | 0·12 | 9/46 (19·6%) | <0·001 | 36/95 (37·9%) |
| Weight for age z-score <-2 | 35/54 (64·8%) | 0·24 | 28/53 (52·8%) | 0·03 | 24/75 (32·0%) | <0·001 | 87/182 (47·8%) |
| Clinical symptoms |  |  |  |  |  |  |  |
| Cough | 65/74 (87·8%) | <0·001 | 61/104 (58·7%) | 0·14 | 38/81 (46·9%) | <0·001 | 164/259 (63·3%) |
| Difficult breathing | 65/74 (87·8%) | 0·82 | 89/104 (85·6%) | 0·002 | 53/81 (65·4%) | 0·001 | 207/259 (79·9%) |
| Fast breathing§§ | 56/74 (75·7%) | 0·61 | 74/104 (71·2%) | 0·004 | 40/81 (49·4%) | 0·001 | 170/259 (65·6%) |
| Chest indrawing | 42/74 (56·8%) | 0·03 | 42/104 (40·4%) | 0·55 | 29/81 (35·8%) | 0·01 | 113/259 (43·6%) |
| Central cyanosis | 0/74 | <0·001 | 16/104 (15·4%) | 0·26 | 18/81 (22·2%) | <0·001 | 34/259 (13·1%) |
| Stridor | 1/74 (1·4%) | 0·40 | 5/104 (4·8%) | 1·00 | 4/81 (4·9%) | 0·37 | 10/259 (3·9%) |
| Inability to drink | 12/74 (16·2%) | 1·00 | 16/104 (15·4%) | 0·005 | 27/81 (33·3%) | 0·02 | 55/259 (21·2%) |
| Lethargy or unconsciousness | 20/74 (27·0%) | 0·06 | 16/104 (15·4%) | <0·001 | 37/81 (45·7%) | 0·02 | 73/259 (28·2%) |
| Fever | 51/74 (68·9%) | 0·22 | 81/104 (77·9%) | 0·86 | 64/81 (79·0%) | 0·20 | 196/259 (75·7%) |
| Oxygen saturation on room air at hospital admission (%) | 93·0 (81·0-98·0); n=43 | 0·02 | 88·0 (80·0-91·0); n=45 | 0·53 | 90·0 (80·0-93·0); n=47 | 0·06 | 89·0 (80·0-94·0); n=135 |
| White blood cell count at diagnosis (cu mm) | 10800 (7850-17 550); n=61 | 0·03 | 8000 (5000-13900); n=43 | 0·40 | 7435 (2625-14012); n=72 | 0·001 | 9350 (5825-14600); n=176 |
| Haemoglobin level at diagnosis (g/dL) | 9·9 (8·0-11·4); n=61 | 0·35 | 10·2 (8·7-11·4); n=40 | 0·07 | 11·1 (9·3-12·6); n=74 | 0·001 | 10·3 (8·6-12·0); n=175 |
| ≥1 other micro-organisms in respiratory sample | 28/84 (33·3%) | 0·76 | 39/110 (35·5%) | 0·21 | 47/105 (44·8%) | 0·14 | 114/299 (38·1%) |
| ≥1 other micro-organisms in other samples | 15/65 (23·1%) | 0·84 | 14/66 (21·2%) | 0·68 | 15/86 (17·4%) | 0·42 | 44/217 (20·3%) |
| Other clinical diagnoses for hospital admission | 65/75 (86·7%) | 0·14 | 57/74 (77·0%) | 0·29 | 58/84 (69·0%) | 0·01 | 180/233 (77·3%) |
| ≥1 sibling <5 years present in household | 19/23 (82·6%) | <0·001 | 6/44 (13·6%) | <0·001 | 38/60 (63·3%) | 0·12 | 63/127 (49·6%) |
| Daycare attendance | 1/4 (25·0%) | 0·17 | 1/41 (2·4%) | <0·001 | 14/44 (31·8%) | 1·00 | 16/89 (18·0%) |

Data are n (%), n/N (%) or median (IQR); n. Statistical comparisons with χ^2^ test using exact p values, Fisher’s exact test or Mann-Whitney U test with p values of less than 0·0167 taken to be significant according to the Bonferroni correction for multiple testing. LMIC=low-income and lower middle-income countries. UMIC=upper middle-income countries. HIC=high-income countries. *Low-income or lower middle-income versus upper middle-income country. †Upper middle-income country versus high-income country. ‡Low-income or lower middle-income country versus high-income country. §Considered absent when missing. §§For age <2 months ≥60 breaths/min; for age 2-11 months ≥ 50 breaths/min; for age 1-5 years ≥ 40 breaths/min.

***Supplemental Table 4*: Other pathogens identified in respiratory samples of children younger than 5 years with influenza-related in-hospital death**

|  | **LMIC** | **UMIC** | **HIC** |
| --- | --- | --- | --- |
| Children with respiratory samples tested for other pathogens | 84/91 | 110/115 | 105/108 |
| Children with ≥1 other micro-organisms present in respiratory sample | 28/84 | 39/110 | 47/105 |
| RSV | 6 | 9 | 13 |
| Parainfluenza | 1 | 3 | 2 |
| Metapneumovirus | 1 | 2 | 0 |
| Adenovirus | 3 | 3 | 4 |
| Rhinovirus | 5 | 8 | 4 |
| S. Pneumoniae | 7 | 4 | 2 |
| H. Influenzae | 4 | 5 | 0 |
| Mycoplasma | 0 | 0 | 0 |
| Enterovirus | 0 | 1 | 1 |
| Bocavirus | 0 | 2 | 1 |
| Coronavirus | 4 | 1 | 1 |
| Pertussis | 0 | 0 | 1 |
| Other | 9 | 19 | 25 |

RSV=Respiratory Syncytial Virus. LMIC=low-income and lower middle-income countries. UMIC=upper middle-income countries. HIC=high-income countries.

***Supplemental Figure 1*: Age distribution at time of influenza-related in-hospital death for children younger than 5 years**

***Supplemental Table 5*: Characteristics of children younger than 5 years with hospital-acquired and community-acquired influenza-related in-hospital death**

|  | **Hospital-acquired (n=38)** | **Community-acquired (n=276)** | **p value** |
| --- | --- | --- | --- |
| Male sex | 15 (39·5%) | 146/275 (52·9%) | 0·12 |
| Age at death (months) | 8·0 (4·9-17·1) | 12·0 (5·2-24·0) | 0·09 |
| <3 months at death | 4 (10·5%) | 33 (12·0%) | 1·00 |
| <6 months at death  Year of death  Age at infection (months)  <3 months at infection | 13 (34·2%)  2015 (2013-2018)  7·4 (3·8-16·4); n=38 8 (21·1%) | 73 (26·4%)  2013 (2011-2017); n=272  11·9 (4·8-25·8); n=212  44 (15·9%) | 0·33  0·01  0·04  0·48 |
| World Bank Income Group |  |  |  |
| LMIC | 4 (10·5%) | 87 (31·5%) | 0·01 |
| UMIC | 20 (52·6%) | 95 (34·4%) | 0·03 |
| HIC | 14 (36·8%) | 94 (34·1%) | 0·86 |
| Type of influenza |  |  |  |
| A H1N1 pre-pandemic | 3 (7·9%) | 7/253 (2·8%) | 0·13 |
| A H1N1 pdm-09 | 11 (28·9%) | 81/253 (32·0%) | 0·72 |
| A H1N2 | 1 (2·6%) | 0/253 | 0·13 |
| A H3N2 | 0 | 29/253 (11·5%) | 0·02 |
| A unsubtyped | 18 (47·4%) | 62/253 (24·5%) | 0·004 |
| B unsubtyped | 9 (23·7%) | 76/253 (30·0%) | 0·45 |
| Other | 0 | 3/253 (1·2%) | 1·00 |
| Comorbidity§ | 36 (94·7%) | 130 (47·1%) | <0·001 |
| Congenital heart disease | 11 (28·9%) | 47 (17·0%) | 0·12 |
| Chronic lung disease | 8 (21·1%) | 26 (9·4%) | 0·05 |
| Genetic disease | 10 (26·3%) | 29 (10·5%) | 0·01 |
| Neurological disease | 7 (18·4%) | 39 (14·1%) | 0·63 |
| Immune disorder | 4 (10·5%) | 14 (5·1%) | 0·25 |
| Prematurity§ | 14 (36·8%) | 27 (9·8%) | <0·001 |
| Gestational age (weeks) | 37·0 (33·5-38·0); n=29 | 38·0 (35·0-39·8); n=72 | 0·05 |
| Intensive care unit (ICU) admission | 37/37 (100·0%) | 171/244 (70·1%) | <0·001 |
| Length ICU admission (days) | 13·0 (7·0-38·0); n=35 | 7·0 (2·0-16·5); n=138 | 0·001 |
| Respiratory support | 36/37 (97·3%) | 160/206 (77·7%) | 0·005 |
| Mechanical ventilation | 33/37 (89·2%) | 113/206 (54·9%) | <0·001 |
| Time between onset of symptoms and death (days) | 14·5 (7·8-37·3) | 12·0 (6·0-21·8); n=212 | 0·14 |
| Time of death relative to seasonality  Death during influenza season | 17/33 (51·5%) | 172/268 (64·7%) | 0·18 |

Data are n (%), n/N (%) or median (IQR); n. §Considered absent when missing.

***Supplemental Table 6*: Clinical and demographic characteristics of children younger than 5 years with community-acquired influenza-related in-hospital death**

|  | **LMICs (n=87)** | **p value*** | **UMICs (n=95)** | **p value†** | **HICs (n=94)** | **p value‡** |
| --- | --- | --- | --- | --- | --- | --- |
| Male sex | 45 (51·7%) | 0·46 | 43 (45·3%) | 0·02 | 58/93 (61·7%) | 0·18 |
| Age at death (months) | 8·0 (4·5-15·6) | 0·03 | 12·0 (4·2-27·6) | 0·07 | 18·0 (9·8-27·3) | <0·001 |
| <3 months at death | 15 (17·2%) | 0·30 | 11 (11·6%) | 0·46 | 7 (7·4%) | 0·07 |
| <6 months at death | 30 (34·5%) | 0·53 | 28 (29·5%) | 0·04 | 15 (16·0%) | 0·006 |
| Year of death  Age at infection (months)  <3 months at infection | 2014 (2011-2018)  7·3 (2·8-16·1); n=60  19 (21·8%) | 0·26  0·01  0·45 | 2013 (2012-2016); n=92  11·6 (4·5-28·2); n=87  16 (16·8%) | 0·01  0·05  0·20 | 2011 (2010-2016); n=93  18·2 (10·1-33·9); n=65  9 (9·6%) | 0·001  <0·001  0·03 |
| Type of influenza |  |  |  |  |  |  |
| A H1N1 pre-pandemic | 3/77 (3·9%) | 1·00 | 3/88 (3·4%) | 0·62 | 1/88 (1·1%) | 0·34 |
| A H1N1 pdm-09 | 12/77 (15·6%) | 0·005 | 31/88 (35·2%) | 0·35 | 38/88 (43·2%) | <0·001 |
| A H1N2 | 0/77 |  | 0/88 |  | 0/88 |  |
| A H3N2 | 6/77 (7·8%) | 0·07 | 16/88 (18·2%) | 0·07 | 7/88 (8·0%) | 1·00 |
| B Yamagata | 0/77 |  | 0/88 |  | 0/88 |  |
| B Victoria | 0/77 |  | 0/88 | 1·00 | 1/88 (1·1%) | 1·00 |
| A unsubtyped | 31/77 (40·3%) | 0·001 | 14/88 (15·9%) | 0·69 | 17/88 (19·3%) | 0·004 |
| B unsubtyped | 25/77 (32·5%) | 0·61 | 25/88 (28·4%) | 1·00 | 26/88 (29·5%) | 0·73 |
| Other | 2/77 (2·6%) | 0·60 | 1/88 (1·1%) | 1·00 | 0/88 | 0·22 |
| Comorbidity§ | 34 (39·1%) | 0·02 | 54 (56·8%) | 0·11 | 42 (44·7%) | 0·46 |
| Prematurity§ | 7 (8·0%) | 0·80 | 9 (9·5%) | 0·64 | 11 (11·7%) | 0·46 |
| Gestational age (weeks)  Length of stay in hospital (days) | 38·0 (34·5-40·0); n=9  7·0 (3·0-12·8); n=80 | 0·87  0·07 | 38·0 (35·0-39·0); n=21  8·0 (4·0-17·0); n=89 | 0·53  0·98 | 38·0 (35·8-40·0); n=42  10·0 (1·0-25·3); n=74 | 0·99  0·16 |
|  |  |  |  |  |  |  |
| Intensive care unit (ICU) admission | 44/80 (55·0%) | 0·44 | 55/89 (61·8%) | <0·001 | 72/75 (96·0%) | <0·001 |
| Length ICU admission (days) | 4·0 (1·0-12·0); n=35 | 0·07 | 9·0 (3·0-17·5); n=33 | 0·43 | 7·0 (1·0-19·5); n=70 | 0·29 |
| ICU not available | 13/80 (16·3%) | <0·001 | 0/88 |  | 0/75 | <0·001 |
| Respiratory support | 37/78 (47·4%) | <0·001 | 49/53 (92·5%) | 0·16 | 74/75 (98·7%) | <0·001 |
| Mechanical ventilation | 14/78 (17·9%) | <0·001 | 31/53 (58·5%) | <0·001 | 68/75 (90·7%) | <0·001 |
| Respiratory support not available | 31/78 (39·7%) | <0·001 | 0/53 |  | 0/75 | <0·001 |
| Time between onset of symptoms and hospital admission (days) | 4·0 (3·0-7·8); n=60 | <0·001 | 2·0 (1·0-4·3); n=90 | 0·58 | 2·0 (1·0-3·0); n=67 | <0·001 |
| Time between onset of symptoms and death (days) | 12·0 (8·0-20·0); n=60 | 0·70 | 12·0 (6·0-22·0); n=87 | 0·77 | 10·0 (5·0-25·0); n=65 | 0·56 |
| Time of death relative to seasonality  Death during influenza season | 61 (70·1%) | 0·16 | 55/92 (59·8%) | 0·54 | 56/87 (64·4%) | 0·52 |

Data are n (%), n/N (%) or median (IQR); n. Statistical comparisons with χ^2^ test using exact p values, Fisher’s exact test or Mann-Whitney U test with p values of less than 0·0167 taken to be significant according to the Bonferroni correction for multiple testing. LMIC=low-income and lower middle-income countries. UMIC=upper middle-income countries. HIC=high-income countries. *Low-income or lower middle-income versus upper middle-income country. †Upper middle-income country versus high-income country. ‡Low-income or lower middle-income country versus high-income country. §Considered absent when missing.

***Supplemental Table 7*: Clinical and demographic characteristics of children younger than 5 years with influenza-related in-hospital death excluding children with missing data for comorbidity or prematurity**

|  | **LMICs (n=23)** | **p value*** | **UMICs (n=63)** | **p value†** | **HICs (n=78)** | **p value‡** |
| --- | --- | --- | --- | --- | --- | --- |
| Male sex | 8 (34·8%) | 0·23 | 32 (50·8%) | 1·00 | 40 (51·3%) | 0·24 |
| Age at death (months) | 9·2 (4·0-23·2) | 0·37 | 12·0 (4·0-28·9) | 0·47 | 14·5 (6·1-28·6) | 0·10 |
| <3 months at death | 5 (21·7%) | 0·29 | 7 (11·1%) | 0·78 | 7 (9·0%) | 0·14 |
| <6 months at death | 8 (34·8%) | 1·00 | 21 (33·3%) | 0·26 | 19 (24·4%) | 0·42 |
| Year of death  Age at infection (months)  <3 months at infection | 2015 (2011-2018); n=23  11·5 (2·5-22·8); n=15  5 (21·7%) | 0·42  0·36  1·00 | 2014 (2012-2017); n=63  12·3 (3·6-28·9); n=60  13 (20·6%) | 0·64  0·37  0·26 | 2014 (2011-2018); n=78  15·1 (6·3-29·7); n=71  10 (12·8%) | 0·33  0·12  0·32 |
| Type of influenza |  |  |  |  |  |  |
| A H1N1 pre-pandemic  A H1N1 pdm-09  A H1N2  A H3N2  B Yamagata  B Victoria  A unsubtyped  B unsubtyped  Other | 0/22  9/22 (40·9%)  0/22  2/22 (9·1%)  0/22  0/22  4/22 (18·2%)7/22 (31·8%)  0/22 | 0·32  0·04  1·00  0·19  0·80  1·00 | 5/62 (8·1%)  10/62 (16·1%)  0/62  7/62 (11·3%)  0/62  0/62  21/62 (33·9%)  22/62 (35·5%)  1/62 (1·6%) | 0·09  0·02  0·38  1·00  0·45  0·26  0·46 | 1/74 (1·4%)  26/74 (35·1%)  0/74  5/74 (6·8%)  0/74  1/74 (1·4%)  20/74 (27·0%)  19/74 (25·7%)  0/74 | 1·00  0·80  0·66  1·00  0·58  0·59 |
| Comorbidity§ | 14 (60·9%) | 0·18 | 48 (76·2%) | 0·26 | 52 (66·7%) | 0·63 |
| Prematurity§ | 8 (34·8%) | 0·42 | 16 (25·4%) | 0·69 | 17 (21·8%) | 0·27 |
| Gestational age (weeks) | 38·0 (34·0-39·0); n=11 | 0·84 | 38·0 (35·0-39·0); n=38 | 0·54 | 38·0 (34·3-40·0); n=52 | 0·95 |
| Length of stay in hospital (days) | 8·0 (4·0-19·0); n=23 | 0·08 | 11·0 (5·0-37·0); n=61 | 0·20 | 12·0 (3·8-25·3); n=78 | 0·47 |
| Intensive care unit (ICU) admission | 20/22 (90·9%) | 0·08 | 43/61 (70·5%) | <0·001 | 76/78 (97·4%) | 0·21 |
| Length ICU admission (days) | 6·0 (1·3-16·8); n=20 | 0·04 | 11·0 (4·0-37·0); n=43 | 0·11 | 8·5 (2·0-21·3); n=74 | 0·39 |
| ICU not available | 0/22 |  | 0/61 |  | 0/78 |  |
| Respiratory support | 11/22 (50·0%) | <0·001 | 59/62 (95·2%) | 0·08 | 78/78 (100%) | <0·001 |
| Mechanical ventilation | 8/22 (36·4%) | 0·01 | 42/62 (67·7%) | <0·001 | 73/78 (93·6%) | <0·001 |
| Respiratory support not available | 8/22 (36·4%) | <0·001 | 0/62 |  | 0/78 | <0·001 |
| Time between onset of symptoms and hospital admission (days) | 2·0 (1·0-7·0); n=15 | 0·33 | 2·0 (0·0-3·8); n=60 | 0·91 | 1·0 (1·0-3·0); n=71 | 0·27 |
| Time between onset of symptoms and death (days) | 13·0 (3·0-23·0); n=15 | 0·18 | 15·5 (7·0-38·8); n=60 | 0·15 | 13·0 (6·0-27·0); n=71 | 0·66 |
| Time of death relative to seasonality  Death during influenza season | 15/23 (65·2%) | 0·33 | 32/63 (50·8%) | 0·07 | 45/67 (67·2%) | 1·00 |

Data are n (%), n/N (%) or median (IQR); n. Statistical comparisons with χ^2^ test using exact p values, Fisher’s exact test or Mann-Whitney U test with p values of less than 0·0167 taken to be significant according to the Bonferroni correction for multiple testing. LMIC=low-income and lower middle-income countries. UMIC=upper middle-income countries. HIC=high-income countries. *Low-income or lower middle-income versus upper middle-income country. †Upper middle-income country versus high-income country. ‡Low-income or lower middle-income country versus high-income country. §Considered absent when missing.

***Supplemental Table 8*: Clinical and demographic characteristics of children younger than 5 years with influenza-related in-hospital death excluding children with influenza A(H1N1)pdm09 virus who died during the pandemic**

|  | **LMICs (n=89)** | **p value*** | **UMICs (n=112)** | **p value†** | **HICs (n=87)** | **p value‡** |
| --- | --- | --- | --- | --- | --- | --- |
| Male sex | 45 (50·6%) | 0·57 | 51 (45·5%) | 0·20 | 48 (55·2%) | 0·55 |
| Age at death (months) | 9·2 (4·6-17·3) | 0·22 | 10·9 (4·2-24·0) | 0·09 | 15·0 (6·7-27·0) | 0·002 |
| <3 months at death | 15 (16·9%) | 0·42 | 14 (12·5%) | 0·36 | 7 (8·0%) | 0·11 |
| <6 months at death | 29 (32·6%) | 0·88 | 35 (31·3%) | 0·11 | 18 (20·7%) | 0·09 |
| Year of death  Age at infection (months)  <3 months at infection | 2015 (2011-2018); n=89  7·6 (2·9-17·4); n=64  19 (21·3%) | 0·32  0·12  0·86 | 2014 (2012-2017); n=109  11·0 (3·8-23·8); n=104  22 (19·6%) | 0·26  0·10  0·17 | 2013 (2011-2018); n=86  15·1 (6·6-27·1); n=73  10 (11·5%) | 0·06  0·002  0·10 |
| Type of influenza |  |  |  |  |  |  |
| A H1N1 pre-pandemic | 3/79 (3·8%) | 1·00 | 5/105 (4·8%) | 0·47 | 2/81 (2·5%) | 0·68 |
| A H1N1 pdm-09 | 12/79 (15·2%) | 0·03 | 31/105 (29·5%) | 0·87 | 23/81 (28·4%) | 0·06 |
| A H1N2 | 0/79 | 1·00 | 1/105 (1·0%) | 1·00 | 0/81 |  |
| A H3N2 | 6/79 (7·6%) | 0·17 | 16/105 (15·2%) | 0·19 | 7/81 (8·6%) | 1·00 |
| B Yamagata | 0/79 |  | 0/105 |  | 0/81 |  |
| B Victoria | 0/79 |  | 0/105 | 0·44 | 1/81 (1·2%) | 1·00 |
| A unsubtyped | 32/79 (40·5%) | 0·02 | 25/105 (23·8%) | 0·50 | 23/81 (28·4%) | 0·13 |
| B unsubtyped | 26/79 (32·9%) | 0·75 | 32/105 (30·5%) | 1·00 | 24/81 (29·6%) | 0·73 |
| Other | 2/79 (2·5%) | 0·58 | 1/105 (1·0%) | 1·00 | 0/81 | 0·24 |
| Comorbidity§ | 35 (39·3%) | 0·001 | 71 (63·4%) | 0·56 | 51 (58·6%) | 0·02 |
| Prematurity§ | 8 (9·0%) | 0·38 | 15 (13·4%) | 0·43 | 16 (18·4%) | 0·08 |
| Gestational age (weeks) | 38·0 (34·0-39·0); n=11 | 0·94 | 38·0 (35·0-39·0); n=37 | 0·82 | 37·0 (34·0-40·0); n=46 | 0·87 |
| Length of stay in hospital (days) | 6·5 (3·0-13·0); n=82 | 0·006 | 9·5 (4·0-19·3); n=106 | 0·95 | 12·0 (3·0-25·3); n=82 | 0·02 |
| Intensive care unit (ICU) admission | 46/81 (56·8%) | 0·09 | 73/106 (68·9%) | <0·001 | 80/83 (96·4%) | <0·001 |
| Length ICU admission (days) | 4·0 (1·5-12·5); n=37 | 0·002 | 10·5 (4·0-27·8); n=50 | 0·13 | 8·0 (2·0-21·5); n=77 | 0·10 |
| ICU not available | 13/81 (16·0%) | <0·001 | 0/106 |  | 0/83 | <0·001 |
| Respiratory support | 39/80 (48·8%) | <0·001 | 65/69 (94·2%) | 0·18 | 82/83 (98·8%) | <0·001 |
| Mechanical ventilation | 16/80 (20·0%) | <0·001 | 46/69 (66·7%) | <0·001 | 76/83 (91·6%) | <0·001 |
| Respiratory support not available | 31/80 (38·8%) | <0·001 | 0/69 |  | 0/83 | <0·001 |
| Time between onset of symptoms and hospital admission (days) | 4·0 (2·0-7·0); n=64 | <0·001 | 2·0 (0·0-4·0); n=107 | 0·85 | 2·0 (1·0-3·0); n=74 | <0·001 |
| Time between onset of symptoms and death (days) | 12·0 (7·0-19·8); n=64 | 0·62 | 12·5 (6·3-24·0); n=104 | 0·80 | 13·0 (5·0-26·0); n=73 | 0·73 |
| Time of death relative to seasonality  Death during influenza season | 63/89 (70·8%) | 0·08 | 63/109 (57·8%) | 0·45 | 48/75 (64·0%) | 0·40 |

Data are n (%), n/N (%) or median (IQR); n. Statistical comparisons with χ^2^ test using exact p values, Fisher’s exact test or Mann-Whitney U test with p values of less than 0·0167 taken to be significant according to the Bonferroni correction for multiple testing. LMIC=low-income and lower middle-income countries. UMIC=upper middle-income countries. HIC=high-income countries. *Low-income or lower middle-income versus upper middle-income country. †Upper middle-income country versus high-income country. ‡Low-income or lower middle-income country versus high-income country. §Considered absent when missing.

***Supplemental Table 9*: Clinical and demographic characteristics of children younger than 5 years with influenza-related in-hospital death excluding children from Ecuador, United Kingdom, Kenya, Turkey and South Africa**

|  | **LMICs (n=65)** | **p value*** | **UMICs (n=26)** | **p value†** | **HICs (n=78)** | **p value‡** |
| --- | --- | --- | --- | --- | --- | --- |
| Male sex | 38 (58·5%) | 0·02 | 8 (30·8%) | 0·17 | 38 (48·7%) | 0·31 |
| Age at death (months) | 7·7 (3·4-14·3) | <0·001 | 23·5 (9·9-39·0) | 0·16 | 15·0 (7·2-28·1) | <0·001 |
| <3 months at death | 13 (20·0%) | 0·06 | 1 (3·8%) | 0·68 | 6 (7·7%) | 0·05 |
| <6 months at death | 24 (36·9%) | 0·08 | 4 (15·4%) | 0·78 | 15 (19·2%) | 0·02 |
| Year of death  Age at infection (months)  <3 months at infection | 2015 (2011-2019); n=65  7·4 (2·6-15·5); n=49  16 (24·6%) | 0·04  <0·001  0·03 | 2013 (2010-2016); n=23  23·0 (11·3-48·5); n=22  1 (3·8%) | 0·54  0·10  0·44 | 2014 (2011-2017); n=77  15·5 (7·6-27·8); n=68  8 (10·3%) | 0·07  <0·001  0·03 |
| Type of influenza |  |  |  |  |  |  |
| A H1N1 pre-pandemic | 3/55 (5·5%) | 0·57 | 0/19 | 1·00 | 2/72 (2·8%) | 0·65 |
| A H1N1 pdm-09 | 14/55 (25·5%) | 0·01 | 11/19 (57·9%) | 0·01 | 19/72 (26·4%) | 1·00 |
| A H1N2 | 0/55 | 0·26 | 1/19 (5·3%) | 0·21 | 0/72 |  |
| A H3N2 | 2/55 (3·6%) | 0·10 | 3/19 (15·8%) | 0·16 | 4/72 (5·6%) | 0·70 |
| B Yamagata | 0/55 |  | 0/19 |  | 0/72 |  |
| B Victoria | 0/55 |  | 0/19 | 1·00 | 1/72 (1·4%) | 1·00 |
| A unsubtyped | 17/55 (30·9%) | 0·13 | 2/19 (10·5%) | 0·08 | 23/72 (31·9%) | 1·00 |
| B unsubtyped | 20/55 (36·4%) | 0·27 | 4/19 (21·1%) | 0·57 | 21/72 (29·2%) | 0·45 |
| Other | 0/55 |  | 0/19 |  | 0/72 |  |
| Comorbidity§ | 20 (30·8%) | 0·06 | 14 (53·8%) | 0·49 | 49 (62·8%) | <0·001 |
| Prematurity§ | 5 (7·7%) | 0·27 | 4 (15·4%) | 1·00 | 14 (17·9%) | 0·09 |
| Gestational age (weeks) | 37·0 (33·8-38·3); n=6 | 0·38 | 33·5 (29·0-38·0); n=4 | 0·16 | 37·0 (34·5-39·0); n=41 | 0·71 |
| Length of stay in hospital (days) | 7·0 (3·0-13·0); n=58 | 0·36 | 9·0 (4·0-15·0); n=23 | 0·43 | 11·0 (3·5-25·0); n=77 | 0·07 |
| (intensive care unit) ICU admission | 25/59 (42·4%) | <0·001 | 22/23 (95·7%) | 1·00 | 75/78 (96·2%) | <0·001 |
| Length ICU admission (days) | 9·5 (5·0-16·8); n=16 | 0·19 | 7·0 (2·0-14·3); n=20 | 0·40 | 8·0 (2·3-20·5); n=72 | 0·57 |
| ICU not available | 13/59 (22·0%) | 0·02 | 0/23 |  | 0/78 | <0·001 |
| Respiratory support | 38/57 (66·7%) | 0·03 | 17/18 (94·4%) | 0·34 | 77/78 (98·7%) | <0·001 |
| Mechanical ventilation | 16/57 (28·1%) | <0·001 | 16/18 (88·9%) | 0·67 | 71/78 (91·0%) | <0·001 |
| Respiratory support not available | 9/57 (15·8%) | 0·10 | 0/18 |  | 0/78 | <0·001 |
| Time between onset of symptoms and hospital admission (days) | 4·0 (2·5-7·5); n=49 | 0·001 | 2·0 (0·0-4·5); n=25 | 0·94 | 2·0 (1·0-3·0); n=69 | <0·001 |
| Time between onset of symptoms and death (days) | 13·0 (8·5-20·0); n=49 | 0·60 | 12·5 (8·0-17·8); n=22 | 0·72 | 12·0 (5·0-24·5); n=68 | 0·77 |
| Time of death relative to seasonality  Death during influenza season | 49/65 (75·4%) | 0·42 | 15/23 (65·2%) | 0·81 | 40/66 (60·6%) | 0·09 |

Data are n (%), n/N (%) or median (IQR); n. Statistical comparisons with χ^2^ test using exact p values, Fisher’s exact test or Mann-Whitney U test with p values of less than 0·0167 taken to be significant according to the Bonferroni correction for multiple testing. LMIC=low-income and lower middle-income countries. UMIC=upper middle-income countries. HIC=high-income countries. *Low-income or lower middle-income versus upper middle-income country. †Upper middle-income country versus high-income country. ‡Low-income or lower middle-income country versus high-income country. §Considered absent when missing.

***Supplemental Table 10*: Clinical and demographic characteristics of children younger than 5 years with
community-acquired, in-hospital influenza-related death (FLU GOLD)* versus community-acquired, in-hospital
RSV-related death (RSV GOLD I)**

|  | **Influenza-related death  (FLU GOLD) (n=276)** | **RSV-related death  (RSV GOLD I)  (n=358)** | **p value** |
| --- | --- | --- | --- |
| Male sex | 146/277 (53·1%) | 190 (53·1%) | 1·00 |
| Age at death (months) | 12·0 (5·2-24·0) | 6·0 (2·5-12·3) | <0·001 |
| <3 months at death | 33 (12·0%) | 91 (25·4%) | <0·001 |
| <6 months at death | 73 (26·4%) | 178 (49·7%) | <0·001 |
| Year of death  LMIC UMIC HIC | 2013 (2011-2017); n=272  87 (31·5%)  95 (34·4%)  94 (34·1%) | 2007 (2003-2011); n=358  117 (32·7%) 155 (43·3%)**  86 (24·0%)** | <0·001  0·41  0·03  0·006 |
| Comorbidity§ | 130 (47·1%) | 183 (51·1%) | 0·32 |
| Prematurity§ | 27 (9·8%) | 66 (18·4%) | 0·002 |
| Gestational age (weeks) | 38·0 (35·0-39·8); n=72 | 38·0 (34·5-40·0); n=140 | 0·79 |
| Length of stay in hospital (days)  Intensive care unit (ICU) available  ICU admission | 8·0 (3·0-16·0); n=243  231/244 (94·7%)  171/244 (70·1%) | 7·0 (3·0-18·5); n=349  267/336 (79·5%)  202/336 (60·1%) | 0·60  <0·001  0·01 |
| Length ICU admission (days) | 7·0 (2·0-16·5); n=138 | 10·0 (4·0-24·0); n=185 | 0·003 |
| Mechanical ventilation^ | 113/206 (54·9%) | 194/329 (59·0%) | 0·37 |
| Time between onset of symptoms and hospital admission (days) | 3·0 (1·0-5·0); n=217 | 3·0 (2·0-6·0); n=251 | 0·02 |
| Time between onset of symptoms and death (days)  Time of death relative to seasonality  Death during season§§ | 12·0 (6·0-21·8); n=212  172/266 (64·7%) | 14·0 (8·0-26·0); n=252  218/277 (78·7%) | 0·008  N/A |

Data are n (%), n/N (%) or median (IQR); n. Statistical comparisons with χ^2^ test using exact p values, Fisher’s exact test or Mann-Whitney U test.
LMIC=low-income and lower middle-income countries. UMIC=upper middle-income countries. HIC=high-income countries.
*In order to ensure comparable populations, we selected children with community-acquired, in-hospital influenza-related death
for this analysis, as these were the inclusion criteria for the RSV GOLD study. **Proportions are different from original
publication because we used World Bank Classifications for 2020. §Considered absent when missing. ^When stratifying
by age, there was no difference in the proportion of children who had been mechanically ventilated (data not shown).
§§For RSV GOLD I, RSV seasonality in the country of origin was reported by collaborators in the questionnaire. For
FLU GOLD, influenza seasonality was not part of the questionnaire and death during influenza season was determined
based on published seasonality data by Li et al. ^18^ We therefore did not compare both groups.

***Supplemental Figure 2*: Age distribution at time of influenza-related and RSV-related death for children younger than 5 years**
